# Continuing Challenges in the Definitive Diagnosis of Cushing’s Disease: A Structured Review Focusing on Molecular Imaging and a Proposal for Diagnostic Work-Up

**DOI:** 10.3390/jcm12082919

**Published:** 2023-04-17

**Authors:** Tessa N. A. Slagboom, Dirk Jan Stenvers, Elsmarieke van de Giessen, Stefan D. Roosendaal, Maartje M. L. de Win, Joseph C. J. Bot, Eleonora Aronica, René Post, Jantien Hoogmoed, Madeleine L. Drent, Alberto M. Pereira

**Affiliations:** 1 Department of Endocrinology and Metabolism, Amsterdam UMC Location Vrije Universiteit Amsterdam, De Boelelaan 1117, 1081 HV Amsterdam, The Netherlands; 2 Pituitary Center Amsterdam, 1105 AZ Amsterdam, The Netherlands; 3 Amsterdam Gastroenterology Endocrinology and Metabolism, 1105 AZ Amsterdam, The Netherlands; 4 Department of Endocrinology and Metabolism, Amsterdam UMC Location University of Amsterdam, Meibergdreef 9, 1105 AZ Amsterdam, The Netherlands; 5 Department of Radiology and Nuclear Medicine, Amsterdam UMC Location University of Amsterdam, Meibergdreef 9, 1105 AZ Amsterdam, The Netherlands; 6 Amsterdam Neuroscience, 1105 AZ Amsterdam, The Netherlands; 7 Department of Radiology and Nuclear Medicine, Amsterdam UMC Location Vrije Universiteit Amsterdam, De Boelelaan 1117, 1081 HV Amsterdam, The Netherlands; 8 Department of (Neuro)Pathology, Amsterdam UMC Location University of Amsterdam, Meibergdreef 9, 1105 AZ Amsterdam, The Netherlands; 9 Department of Neurosurgery, Amsterdam UMC Location University of Amsterdam, Meibergdreef 9, 1105 AZ Amsterdam, The Netherlands; 10 Cancer Center Amsterdam, 1081 HV Amsterdam, The Netherlands

**Keywords:** Cushing’s disease, PET, diagnosis, molecular imaging, hypercortisolism, pituitary

## Abstract

The definitive diagnosis of Cushing’s disease (CD) in the presence of pituitary microadenoma remains a continuous challenge. Novel available pituitary imaging techniques are emerging. This study aimed to provide a structured analysis of the diagnostic accuracy as well as the clinical use of molecular imaging in patients with ACTH-dependent Cushing’s syndrome (CS). We also discuss the role of multidisciplinary counseling in decision making. Additionally, we propose a complementary diagnostic algorithm for both de novo and recurrent or persistent CD. A structured literature search was conducted and two illustrative CD cases discussed at our Pituitary Center are presented. A total of 14 CD (*n* = 201) and 30 ectopic CS (*n* = 301) articles were included. MRI was negative or inconclusive in a quarter of CD patients. ^11^C-Met showed higher pituitary adenoma detection than ^18^F-FDG PET–CT (87% versus 49%). Up to 100% detection rates were found for ^18^F-FET, ^68^Ga-DOTA-TATE, and ^68^Ga-DOTA-CRH, but were based on single studies. The use of molecular imaging modalities in the detection of pituitary microadenoma in ACTH-dependent CS is of added and complementary value, serving as one of the available tools in the diagnostic work-up. In selected CD cases, it seems justified to even refrain from IPSS.

## 1. Introduction

Cushing’s syndrome (CS) is a rare condition characterized by prolonged increased exposure to cortisol, leading to a classic clinical appearance that is accompanied by multiple comorbidities. The most prevalent cause of Cushing’s syndrome is excess exogenous cortisol as a consequence of glucocorticoid use. When exogenous Cushing’s syndrome is excluded, screening for endogenous cortisol is warranted. For this purpose, several screening tests are available. In the case of the biochemical confirmation of hypercortisolism, the measurement of ACTH concentrations is pivotal to determine whether the Cushing’s syndrome is ACTH-dependent or not. Low ACTH values are indicative of an adrenal source of hypercortisolism, while normal or high ACTH (“ACTH-dependent CS”) points to a pituitary (“Cushing’s disease”, CD) or ectopic source (“ectopic Cushing’s syndrome”, ECS). Since pituitary adenoma (CD) forms the majority (approx. 70%) of the cases with ACTH-dependent CS, a pituitary MRI is recommended [1]. However, previous studies have shown a negative or inconclusive scan in a considerable number of these patients [1,2,3,4].

Until recently, it was advocated to perform subsequent bilateral inferior petrosal sinus sampling (IPSS) when MRI results were negative, inconclusive, or showed a microadenoma (<10 mm), and IPSS is considered the golden standard to distinguish CD from ECS. In 2003, a consensus statement on the diagnosis of Cushing’s syndrome described, for the first time, the possibility of refraining from a further evaluation using IPSS [5] in case of an adenoma with a diameter of more than 6 mm in combination with the clinical suspicion and dynamic biochemical confirmation of a pituitary source [5]. The cut-off diameter of 6 mm was based on studies from the 1990s, reporting a considerable number of pituitary lesions of unknown significance (“incidentalomas”) and artifacts in the general population using 1.5-Tesla MRI. Incidentalomas usually did not exceed 6 mm in diameter [6,7,8]. The previously proposed cut-off diameter to distinguish ACTH-producing pituitary adenomas (corticotropinoma) from pituitary incidentalomas in ECS was confirmed in a more recent study by Yogi-Morren et al., showing 96% specificity for a cut-off diameter of 6 mm [9]. According to a recent update of the international diagnostic guideline, CD can be presumed when biochemical tests are in line with a pituitary source of ACTH secretion in the presence of a pituitary macroadenoma (≥10 mm). In these cases, there is a consensus that no further IPSS is justified [10]. In the case of a microadenoma smaller than 6 mm, there still is a strong recommendation to perform IPSS. Expert opinions differ on recommendations for the indication of IPSS in the case of a microadenoma between 6 and 9 mm. The majority (up to 90%) of CD adenomas are microadenomas; therefore, invasive diagnostic modalities are currently recommended in most patients [11,12].

Moreover, IPSS is currently recommended for CD patients with persistent disease after surgery (approximately a quarter of the patients) and without histological confirmation of a corticotroph adenoma [10,13]. Since long-term recurrence rates of CD vary between 15–44%, lifelong follow-up is strongly advised, and IPSS can also be reconsidered as a diagnostic tool in selected cases of CD recurrence without previous histological confirmation [10,14,15,16].

Centers vary in their institutionally based experience with diagnostic (both biochemical and IPSS) modalities for ACTH-dependent hypercortisolism as well as in the used cut-off values. Moreover, the decreasing availability of corticotropin-releasing hormone (CRH) worldwide also hampers the possibility of performing a CRH stimulation test, or CRH stimulation during IPSS. In addition, the availability of an experienced multidisciplinary team consisting of endocrinologists, neurosurgeons, (nuclear and intervention) radiologists, and pathologists can shift the diagnostic approach in CS from a static protocol-based approach to a more personalized approach.

Newly available imaging techniques with increased accuracy (both structural and molecular) may substitute IPSS in some cases. Bashari et al. recently proposed a stepwise approach to optimize the MRI protocol with additional sequences and magnetic field strengths to improve adenoma detection [17]. When improved structural imaging modalities still fail to detect a pituitary adenoma, the next diagnostic consideration could be molecular imaging, enabling a combination of anatomical (computed tomography, CT/magnetic resonance imaging, and MRI) with functional (metabolic) tissue information (positron emission tomography and PET). The results, though still based on small patient cohorts, are promising [17,18].

However, (interpretation of) these new diagnostic (imaging) techniques are not yet available in a substantial part of referral centers, and differences in available diagnostic capabilities may lead to different strategies between (referral) centers.

This study aimed to provide a structured analysis of the diagnostic accuracy as well as the clinical use of molecular imaging in patients with ACTH-dependent CS. We also discuss the role of multidisciplinary counseling within diagnostic decision making. Additionally, we propose a complementary diagnostic algorithm, both for de novo and recurrent or persistent CD.

## 2. Materials and Methods

### 2.1. Search Strategy and Selection Criteria

A structured PubMed search was conducted in January 2023 to collect relevant studies describing molecular imaging in ACTH-dependent CS using the following terms: “positron emission tomography” AND “Cushing”. This resulted in 199 articles. After the screening of titles and abstracts, 143 articles were excluded. Exclusion criteria were abstracts, poster presentations, case reports, reviews, meta-analyses, and articles written in languages other than English. The remaining 56 articles were screened for eligibility criteria—we only included peer-reviewed, original prospective, and retrospective cohort studies. Since some of the found studies were from the same research group, we checked if duplication of cohorts was described. If not, the studies were included. In total 33 articles could be included after this process. Hereafter, we searched within these 33 included articles for additional relevant references, and were able to include 8 additional articles for review, leading to a total of 41 articles.

### 2.2. Data Extraction

Data extraction included the following variables: study (year, research group, and design), population (number, age, sex, pituitary adenoma size or tumor with ectopic ACTH secretion), imaging modalities (including used sequences and magnetic field strength), tracer (including dosage), data and results (true positive, true negative, false negative, and false positive; sensitivity and specificity), and the conclusions of authors.

### 2.3. Data Analysis and Synthesis

Descriptive data per included study were presented in tables. Data were synthesized in diagnostic accuracy tables, giving the total amount and percentage of true positives, false positives, true negatives, and false negatives for different PET tracers.

### 2.4. Illustrative Patient Cases

Two illustrative CD patient cases, which were discussed at the Amsterdam Pituitary Center multidisciplinary team (MDT) meeting and thereafter underwent molecular imaging, are presented to emphasize the value of proposed complementary diagnostic algorithms.

## 3. Results

### 3.1. Molecular Imaging in Cushing’s Disease (See Also Table 1)

The use of molecular imaging in Cushing’s disease was described in a total of 201 patients in 14 articles between 2006 and 2022, including 6 prospective (of which 1 as a pilot) and 8 retrospective cohorts, as shown in Table 1. Tracers included ^18^F-fluorodeoxyglucose (^18^F-FDG, eight articles including one with and without CRH stimulation), ^11^C-methionine (^11^C-Met, five articles), ^68^Gallium-DOTA-TATE (^68^Ga-DOTA-TATE, one article), ^68^Gallium-pentixafor (^68^Ga-pentifaxor, one article), ^68^Gallium-DOTA-CRH (^68^Ga-DOTA-CRH, one article), ^18^F-fluorethyltyrosine (^18^F-FET, one article), and ^13^N-ammonia (one article). Functional imaging modalities combined with MRI were PET in four articles, PET–CT in eight articles, and PET–MR in two articles. We did not include studies describing octreotide/single-photon emission computerized tomography (SPECT) because of their inferior spatial resolution compared to PET. Concerning the magnetic field strength of MRI in these 14 studies; 6 used 1.5-Tesla, 4 used 3.0-Tesla, 1 did not use MRI, and, for 4 studies, the MRI field strength was unknown. A total of 156 (78%) de novo and 45 (22%) recurrent cases of CD were reviewed. Age ranged between 11 and 77 years, and a female sex preponderance was found (136 females, 57 males, and unknown for 8). Of the pituitary adenomas, MRI was negative or inconclusive in approximately a quarter of patients (55/194 = 28%, with a similar percentage when including only an MRI sequence with presumed higher sensitivity [spoiled gradient recalled echo sequence, SPGR]: 23/94 = 24%). Adenoma size was unknown for 51/201 (25%) patients. Of the remaining 150 adenomas described in the studies, 39 (26%) were macroadenoma and 111 (74%) were microadenomas. Within the microadenoma group, adenoma size was not further specified in 56/111 (50%), 35/111 (32%) were sized ≤6 mm, and 20/111 (18%) were sized 7 to 9 mm.

**Table 1:** Original studies on molecular imaging in Cushing’s disease.

Ref.	Author	Year	Tracer	Imaging Modality	Population				MRI Findings				Results	Conclusion Authors
					N	Age	Sex *	De Novo/Recurrent	Negative or Inconclusive	≤6 mm	7–9 mm	≥10 mm		
[19]	Tang et al. (Erasme Hospital, Belgium)	2006	^11^C-Met (555 Mbq)	PET + MRI (1.5T SE, gadolinium-based)	8 **	X	X	0/8	7/8 (88%)	1/8 (13%)			Population: pituitary adenoma with biochemical evidence of active residual/regrowth ≥ 3 mnd post-TSS Adenoma detection: - MRI = 1/8 (13%) 1/1 correct localization; 7/8 not able to differentiate residual adenoma from scar formation - ^11^C-Met PET = 8/8 (100%), 8/8 correct localization Histological confirmation: unknown Outcome: - 6/8 GKRS: 4/6 no medical Tx needed after GKRS, 2/6 ketoconazole - 1/8 s TSS - 1/8 observation	^11^C-Met -PET is a sensitive technique complementary to MRI for the detection of residual or recurrent pituitary adenoma. The metabolic data provides decisive complementary information for dosimetry planning in GKRS (particularly ACTH-secreting pituitary adenoma). It should gain a place in the efficient management of these tumors.
[20]	Alzahrani et al. (King Faisal Specialist Hospital and Research Centre, Saudi Arabia)	2009	^18^F-FDG (370 Mbq)	PET–CT + MRI (1.5T SE, gadolinium-based)	12	40 (31–51)	33%	7/5	4/12 (33%)	2/12 (17%)	3/12 (25%)	3/12 (25%)	Population: proven CD on biochemical (stimulation) tests, radiological and/or histopathological findings Adenoma detection: - MRI = + in 8/12 (67%), − in 4/12 (33%); of which 1/4 + on PET–CT - ^18^F-FDG PET–CT = + in 7/12 (58%) and - in 5/12 (42%) of which 2/5 (40%) + on MRI Not histologically proven (*n* = 2): negative on MRI and ^18^F-FDG PET–CT, IPSS: CD, 1/2 clear lateralization. De novo (*n* = 7): - MRI = + in 4/7 (57%) - ^18^F-FDG PET–CT = + in 3/7 (43%) but all seen on MRI Recurrent (*n* = 5) - MRI = + in 4/5 (80%) - ^18^F-FDG PET–CT = + in 4/5 (80%) of which 1 not seen on MRI ^18^F-FDG PET–CT to size negative MRI = 1/4 (25%) ≤6 mm = 2/2 (100%) 6–9 mm = 2/3 (67%) ≥10 mm = 2/3 (67%) Histological confirmation: yes in *n* = 10, no in *n* = 2 (IPSS) Outcome after TSS: - negative MRI: 1/4 remission, 2/4 persistent, 1/4 controlled with Tx - ≤6 mm: 2/2 persistent - 6–9 mm: 2/3 persistent, 1/3 remission - ≥10 mm: 2/3 remission, 1/3 persistent Without histological confirmation: 1/2 persistent, 1/2 controlled with Tx. De novo: 3/7 remission, 3/7 persistent, 1/7 controlled with Tx Recurrent: 4/5 persistent, 1/5 remission	^18^F-FDG PET–CT is positive in ± 60% of CD cases. Although the majority of cases with positive ^18^F-FDG PET–CT had positive MRI, PET–CT may detect some cases with negative MRI and thus provides important diagnostic information. If these findings are confirmed in larger studies, PET–CT might become an important diagnostic technique, especially when MRI is negative or if IPSS is not available or inconclusive
[21]	Ikeda et al. (Southern Tohuku General Hospital, Japan)	2010	^18^F-FDG (185 Mbq) + ^11^C-Met (280–450 Mbq) FDG 1 h after Met injection	PET-MRI (12 by 3.0T) + MRI (19 by 3.0T, 16 by 1.5T; SE and gadolinium-based)	35	46.5 (11–76)	29%	35/0	10/30 (33%) ***	30/35 (86%) of which 18/30 overt (60%) 12/30 preclinical (40%)		5/35 (14%) of which 2/5 overt (40%) 3/5 preclinical (60%)	Population: histologically proven Cushing’s adenoma after TSS, overt (*n* = 20) and preclinical (*n* = 15) Adenoma detection: - MRI microadenoma (*n* = 30): - 1.5T = + in 8/14 (57%) of which preclinical: + in 2/5, overt: + in 6/9 - 3.0T = + in 4/16 (25%) of which preclinical: + in 1/7, overt: + in 3/9 12/30 (40%): good correlation to surgical findings: 10 false-, 6 false+ and 3 double pituitary adenoma - ^11^C-Met-PET/MRI 3.0T = + in 11/11 (100%) and 100% accuracy - ^18^F-FDG–PET/MRI 3.0T = + in 8/12 (67%) To size - microadenoma: ^11^C-Met-PET/MRI: + in 8/8 ^18^F-FDG–PET/MRI: + in 6/8 - macroadenoma: ^11^C-Met-PET/MRI: + in 3/3 ^18^F-FDG–PET/MRI: + in 2/3 To stage - preclinical: ^11^C-Met-PET/MRI: + in 5/5 ^18^F-FDG–PET/MRI: + in 2/5 - overt: ^11^C-Met-PET/MRI: + in 6/6 ^18^F-FDG–PET/MRI: + in 6/7 Histological confirmation: yes Outcome: unknown	^11^C-Met-PET/3.0T MRI provides higher sensitivity for determining the location and delineation of Cushing’s adenoma than other neuroradiological imaging techniques ([dynamic] MRI and CT). A pituitary adenoma is better delineated on ^11^C-Met -PET than ^18^F-FDG–PET. No difference in SUVmax of ^11^C-Met and ^18^F-FDG -PET between overt and preclinical CD in terms of glucose and amino acid metabolism within adenoma; therefore, ^11^C-Met-PET/MR imaging is useful in detecting early-stage Cushing’s adenoma. If there is PET-positive imaging around the pituitary region and CD is doubted endocrinologically, then we believe that surgery is justified, implying that IPSS can be omitted.
[22]	Seok et al. (Yonsei University College of Medicine, Korea), Prospective	2013	^18^F-FDG (259–333 Mbq)	PET + MRI (1.5T SE, gadolinium contrast)	2 **	17 + 58	50%	2/0	1/2 (50%)	0	0	1/2 (50%)	Population: 32 patients investigated for pituitary lesions Adenoma detection: - MRI: + in 1/2 (50%; macroadenoma) - ^18^F-FDG–PET: + in 1/2 (50; macroadenoma; same as MRI) Histological confirmation: unknown Outcome: unknown	^18^F-FDG–PETis an ancillary tool for detecting and differentiating various pituitary lesions in certain circumstances. Further PET studies determining the right threshold of SUVmax or conjugating various tracer molecules will be helpful.
[23]	Chittiboina et al. (National Institute of Neurological Diseases and Stroke, USA) Prospective	2015	^18^F-FDG (≥ 18 years = 370 Mbq and <18 years = 2.96 Mbq/kg)	hrPET + MRI (1.5T SE + SPGR, gadolinium contrast)	10	30.8 ± 19.3 (11–59)	30%	9/1	SE: 6/10 (60%), SPGR: 3/10 (30%)	7/10 (70%) (max diameter of adenoma at surgery)	0/10	3/10 (30%) (max diameter of adenoma at surgery)	Population: consecutive patients with CD (biochemical tests, MRI—pituitary, and/or IPSS) Adenoma detection: - MRI SE = + in 4/10 (40%) SPGR = + in 7/10 (70%), - ^18^F-FDG hrPET = + in 4/10 (40%) 3 detected on SPGR but not on PET 1 detected on SE but not on PET 2 detected on PET but not on SE Location corresponded with the surgical location in all positive MRI and hrPET. De novo (*n* = 9) MRI SE: + in 4/9 MRI SPGR: + in 6/9 ^18^F-FDG hrPET: + in 4/9 Recurrent (*n* = 1): MRI SE: + in 0/1 MRI SPGR: + 1/1 ^18^F-FDG hrPET: + in 0/1 To size: - negative SPGR: 0/3 + on PET - negative SE: 2/6 + on PET - ≤6 mm: 2/7 - on all; 2/7 + on SE + SPGR, - on PET; 2/7 + on PET + SPGR, - on SE 1/7 + on SPGR, - on PET + SE - ≥10 mm: 2/3 + on all 3, 1/3 - on all 3 Histological confirmation: yes Outcome: 10/10 (100%) biochemical remission after surgery.	While ^18^F-FDG hrPET can detect small functioning corticotropinomas (3 mm) and is more sensitive than SE MRI, SPGR MRI is even more sensitive. High midnight ACTH levels and an attenuated response to CRH stimulation can predict ^18^F-FDG hrPET-positive adenomas in CD.
[24]	Boyle et al. (National Institute of Neurological Diseases and Stroke, USA) Prospective	2019	^18^F-FDG (≥18 years = 370 Mbq and <18 years= 2.96 Mbq/kg) with and without CRH stimulation (1 mcg/kg): 0, 2, or 4 h prior to PET	hrPET + MRI (SE + SPGR, gadolinium contrast)	27	34.9 ± 16.8 (10–61)	26%	23/4	9/27 (33%) of which 5/9 negative (-) and 4/9 questionable (?)	7/27 (26%)	3/27 (11%)	8/27 (30%)	Population: subjects with likely diagnosis of CD (based on biochemical data, IPSS when incongruent or MRI negative/lesions <6 mm) Adenoma detection: (2 reviewers: neuroradiologists) - MRI = + in 18/27 (67%), −/? in 9/27 of which 2/5+ on PET [1 after CRH] - ^18^F-FDG hrPET no CRH = ≥1 reviewer + in: 12/27 (44%) both reviewers: + in 8/27 (30%) - in 15/27(56%): of which 8 + on MRI - ^18^F-FDG hrPET PET with CRH = ≥ 1 reviewer + in: 15/27 (56%) both reviewers: + in 14/27 (52%) - in 12/27(44%): of which 5 + on MRI No false+ De novo (*n* = 23) - MRI = + in 17/23 (74%) - ^18^F-FDG hrPET no CRH = ≥1 reviewer + in: 11/23 (48%) both reviewers: + in 7/23 (30%) - ^18^F-FDG hrPET with CRH = ≥1 reviewer + in: 14/23 (61%) both reviewers: + in 11/23 (48%) Recurrent (*n* = 4) - MRI = + in 1/4 (25%), −/? in 3/4 - ^18^F-FDG hrPET no CRH = ≥1 reviewer + in: 1/4 (25%) both reviewers: + in 1/4 (25%) same + as on MRI - ^18^F-FDG hrPET with CRH = ≥1 reviewer + in: 2/4 (50%) both reviewers: + in 2/4 (50%) same + as on MRI and 1 - MRI To size: - inconclusive (*n* = 9): ^18^F-FDG hrPET no CRH = + in 1/9 ^18^F-FDG hrPET with CRH = + in 2/9 - <6 mm (*n* = 7): ^18^F-FDG hrPET no CRH = + in 3/7 ^18^F-FDG hrPET with CRH = + in 4/7 - 7–9 mm (*n* = 3) ^18^F-FDG hrPET no CRH = + in 3/3 ^18^F-FDG hrPET with CRH = + in 3/3 - ≥10 mm (*n* = 8) ^18^F-FDG hrPET no CRH = + in 5/8 ^18^F-FDG hrPET with CRH = + in 7/8 Histological confirmation: yes Outcome: unknown	CRH stimulation may lead to increased ^18^F-FDG uptake and an increased rate of detection of corticotropinomas in CD. These results also suggest that some MRI-invisible adenomas may be detectable by CRH-stimulated ^18^F-FDG–PET imaging. These findings invite further prospective evaluation; if validated, CRH-stimulated PET imaging could complement MRI to improve the presurgical visualization of ACTH-secreting microadenomas.
[25]	Koulouri et al. (Wellcome-MRC Institute of Metabolic Science)	2015	^11^C-Met (300–400 Mbq)	PET–CT + MRI (1.5T SE + SPGR, gadolinium contrast)	18	43 (17–77)	20%	10/8	SPGR: 3/18 (17%) SE: 7/18 (39%)	X (de novo: 6/10 possible microadenoma on conventional MRI SE)			Population: ACTH-dependent CS, de novo and residual/recurrent (all TSS + 2 also previous Rx) Adenoma detection: - De novo: MRI SE = + in 6/10 (60%) MRI SPGR = + in 10/10 (100%) ^11^C-Met PET–CT: + in 7/10 (70%); all 7 co-localized with adenoma on SPGR − in 3/10 (30%) - Recurrent: MRI SE/SPGR: + in 5/8 ^11^C-Met PET–CT: + in 5/8 (63%); all 5 co-localized with adenoma on MRI − in 3/8 (38%), all 3 also - on MRI Histological confirmation.: yes Outcome after TSS: De novo: described for 6/10: + on PET: 3/7 remission, 4/7 not given − on PET:1/3 remission, 2/3 persistent Recurrent: described for 2/8: 2/2 in remission	^11^C-Met PET/MRI may help inform decision making in: (i) de novo CD and suspected lesion on MRI (SE, dynamic, or SPGR) to confirm functionality within the visualized lesion; (ii) after noncurative TSS or with the recurrent disease when further surgery or Rx is considered: to distinguish the disease from post-treatment change/scar tissue. We speculate that a multimodel pituitary imaging approach using SE and/or SPGR pituitary MRI and ^11^C-Met PET–CT could be adopted for ‘difficult’ pituitary Cushing’s cases in order to maximize the chance of adenoma detection and localization.
[26]	Feng et al. (The First Affiliated Hospital, Sun Yat-sen University, China)	2016	^11^C-Met (280–450 Mbq) + ^18^F-FDG (370 Mbq) Preformed on separate days within 1 week	PET–CT + MRI	15 **	38.3 ± 9.19 (28–55)	47%	11/4	2/15 (13%) *** (2 equivocal MRI: 4 + 5 mm)	6/15 (40%)	5/15 (33%)	4/15 (27%)	Population: adenoma location in functional PA Adenoma detection: - MRI: + in 13/15 (87%), equivocal in 2/15 (13%) - ^18^F-FDG PET–CT: + in 10/15 (67%), in 5/15 (33%), no false+, 100% specificity (all concordant) - ^11^C-Met PET–CT: + in 15/15 (100%), of which 1/15 false +, specificity: 93% De novo - MRI: + in 10/11, - in 1/11: + on both FDG and Met, but disconcordant (5 mm) - ^18^F-FDG PET–CT: + in 9/11 (82%), - in 2/11: 4 and 5 mm - ^11^C-Met PET–CT: + in 11/11 (1 false +) Recurrent - MRI: + in 3/4, − in 1/4: + on both FDG and Met (4 mm) - ^18^F-FDG PET–CT: + in 1/4, - in 3/4 - ^11^C-Met PET–CT: + in 4/4 To size: - equivocal (*n* = 2): 2/2 + on FDG and PET, but 1/2 disconcordant: lesion of FDG was a true lesion during surgery - <6 mm (*n* = 6): ^18^F-FDG + in 3/6 ^11^C-Met + in 6/6 of which 1 false + - 6–9 mm (*n* = 5): ^18^F-FDG + in 4/5 ^11^C-Met + in 5/5 - ≥10 mm (*n* = 4): ^18^F-FDG + in 3/4 ^11^C-Met + in 4/4 Histological confirmation: yes Outcome: unknown	The positive rate of ^11^C-Met PET–CT in ACTH-secreting pituitary adenoma is as high as 100% and a promising, noninvasive method that could even replace IPSS under specific circumstances. The sensitivity of ^18^F-FDG PET–CT is unsatisfactory. Functional pituitary adenoma in general: PET–CT may be useful to detect tumors in patients with equivocal MRI results. Met–PET can provide valuable diagnostic information when ^18^F-FDG–PET yields negative results (not vice versa).
[27]	Wang et al. (The First Affiliated Hospital, Sun Yat-sen University, China) Prospective	2019	^13^N-ammonia (444–592 Mbq) + ^18^F-FDG (370 Mbq) FDG 2 h after ammonia	PET–CT + MRI (3.0T SE, gadolinium contrast)	10 **	38.4 ± 9.55 (28–55)	40%	8/2	1/10 (10%) ***	3/10 (30%)	2/10 (20%)	5/10 (50%)	Population: = position of pituitary tissue in patients with pituitary adenoma Adenoma detection: - MRI = + in 9/10 (90%), also correct localization − in 1/10 (10%); de novo, 5 mm + on ^18^F-FDG but - on ^13^N-ammonia - ^13^N-ammonia PET–CT: + in 9/10 (90%), also correct localization - in 1/10 (10%); same as - on MRI All positives concurrent L/R position. Histological confirmation.: yes Outcome: unknown	Pituitary adenoma in general: ^13^N-ammonia PET–CT imaging is a sensitive means for locating and distinguishing pituitary tissue from PAs, particularly those with tumor maximum diameter <2 cm. It is potentially valuable in the detection of pituitary tissue in pituitary adenoma.
[28]	Zhou et al. (Ruijin Hospital, China)	2019	^18^F-FDG (4.44–5.55 Mbq/kg)	PET–CT + MRI	11	44.8 ± 14.7 (17–74)	34%	11/0	4/11 (36%)	4/11 (36%)		3/11 (27%)	Population: patients who underwent whole-body ^18^F-FDG PET–CT to identify ACTH-dependent CS source Adenoma detection: - MRI: + in 7/11 (64%), specificity: 72% - ^18^F-FDG PET–CT: + 4/11 (36%, all also seen on MRI), specificity: 50% To size: - inconclusive: ^18^F-FDG + in 0/4 - microadenoma: ^18^F-FDG + in 3/4 - macroadenoma: ^18^F-FDG + in 1/3 Histological confirmation: yes Outcome: unknown	^18^F-FDG PET–CT plays a role in localizing the site for EAS (especially mediastinal, pancreatic, and nasal endocrine tumors), although it plays a limited role in CD
[29]	Walia et al. (Post Graduate Institute of Medical Education and Research, India) Prospective	2020	^68^Ga-DOTA-CRH (111–185 Mbq)	PET–CT + MRI (3.0T SPGR, gadolinium contrast)	24	37.4 (13–64)	37%	24/0	4/24 (17%) ***	10/24 (42%)	7/24 (29%)	7/24 (29%)	Population: ACTH-dependent CS Adenoma detection: - MRI: + in 20/24 (83%), − in 4/24 (17%; of which 1 empty sella, 2 normal and 1 post-op changes > CRH PET–CT correctly delineated lesions) - ^68^Ga-DOTA-CRH PET–CT = + in 24/24 (100%), lateralization also 100% Histological confirmation.: yes Outcome: PET information used for intraoperative navigation: biochemical remission in 12/17 (71%) of micro- and 4/7 (57%) macroadenoma	^68^Ga-CRH PET–CT is targeting CRH receptors that not only delineate corticotropinoma and provide the surgeon with valuable information for intraoperative tumor navigation but also help in differentiating a pituitary from an extra-pituitary source of ACTH-dependent CS.
[30]	Berkman et al. (Kantonsspital Aarau, Switzerland)	2021	^11^C-Met (200 Mbq) + ^18^F-FET (300 Mbq)	PET-MRI (3.0T SPGR, contrast imaging)	15	47.2 (18–69)	7%	12/3	4/15 (27%) ***	15/15 (100%), mean tumor volume = 0.07		0	Population: patients who underwent TSS for biochemically proven CD Adenoma detection: - MRI: + in 11/15 (73%) - ^11^C-Met PET-MRI: + in 9/9 (100%) of which 1 suggested lesion contralateral to actual lesion during surgery: PPV = 8/9 (89%) - ^18^F-FET-PET: + in 9/9 (100%), PPV localization = 9/9(100%) Histological confirmation: yes Outcome: Initial biochemical remission in 13/15 (87%). Recurrence in 2/15 during follow-up (1 ^18^F-FET and 1 ^11^C-Met/^18^F-FET): recurrence rate ^18^F-FET = 20% and ^11^C-Met = 50%. Non-remission rate: 1 (17%) of ^18^F-FET and 1 (33%) ^11^C-Met/^18^F-FET group	Preoperative hybrid ^18^F-FET-PET/MRI and ^11^C-Met-PET/MRI have a high predictive value in localizing corticotroph adenoma selective for adenomectomy in CD. Even with a limited number of patients investigated in this study, the performance of ^18^F-FET PET/MRI for localizing microadenoma may encourage validating studies and, thereafter, more widespread use, to give more patients access to a potentially effective, and, in terms of selectivity, a less detrimental surgical therapy option.
[31]	Novruzov et al. (University College London Hospital, UK)	2021	^68^Ga-DOTA-TATE (120–200 Mbq)	PET–CT	7 **	48 ± 17 (26–68)	43%	0/7	X				Population: consecutive patients with suspected pituitary pathology referred for ^68^Ga-DOTA-TATE PET–CT Adenoma detection: 9 suspected recurrent CS: - ^68^Ga-DOTA-TATE PET–CT: pituitary + in 7/9 (78%), − in 1/9 (occult) and 1/9 uptake pancreas Pituitary uptake: 7/7 in recurrent CD 0/2 in ECS Histological confirmation.: yes Outcome: unknown	Recurrent CS is associated with positive pituitary uptake of ^68^Ga-DOTA-TATE. Although in these cases it would not be possible to distinguish pathological from physiological uptake, positive ^68^Ga-DOTA-TATE is useful as it indicates the presence of functioning pituitary tissue. The absence of pituitary uptake in patients with recurrent CS suggests an ectopic ACTH source.
[32]	Ding et al. (Peking Union Medical College Hospital, China) Prospective	2022	^68^Ga-pentixafor (111–185 MBq) + ^18^F-FDG (5.55 MBq/kg)	PET–CT + MRI (rapid dynamic contrast-enhanced)	7 **	38.0 ± 9.5	14%	4/3	3/7 (43%) ***	7/7 (100%), M ± SD = 5.9 ± 2.9 mm		0	Population: Cushing’s syndrome who underwent ^68^Ga-pentixafor Adenoma detection: - MRI: + in 4/7 (57%) -^68^Ga-pentixafor PET–CT: + in 6/7(86%) - ^18^F-FDG PET–CT: + in 1/7 (14%) De novo - MRI: + in 4/4 (100%) - ^68^Ga-pentixafor PET–CT: + in 4/4 - ^18^F-FDG PET–CT: + in 1/4 Recurrent - MRI: + in 0/3 - ^68^Ga-pentixafor PET–CT: + in 2/3 - ^18^F-FDG PET–CT: + in 0/3 Histological confirmation: unknown Outcome: unknown	^68^Ga-pentixafor PET–CT is promising in the differential diagnosis of both ACTH-independent and ACTH-dependent CS. The ACTH-pituitary adenoma detection rate of ^68^Ga-pentifaxor PET–CT was greater than that of contrast-enhanced MRI of ^18^F-FDG PET–CT.

^11^C-Met = ^11^C-Methionine; ^18^F-FDG = ^18^F-Fluorodeoxyglucose; ^68^Ga = ^68^Gallium; AD = adenoma; ECS = ectopic source; ^18^F-FET = 18-F-fluoroethyl-L-tyrosine; GKRS = gamma-knife radiosurgery; HDDST = high dose dexamethasone suppressive tests; hrPET = high-resolution PET; N/R = de novo/recurrent or persistent; SE = spin echo; SPGR = spoiled gradient recalled sequences; TSS = transsphenoidal surgery; X = unknown; * = given as % male, ** = corticotropinoma out of total cohort, *** = based on MRI results, not clear how adenoma size is calculated.

Diagnostic accuracy: Table 2 shows the diagnostic accuracies for pituitary adenoma detection in CD for the different tracers. The only tracers that were studied multiple times were ^18^F-FDG and ^11^C-Met. While the overall sensitivity of adenoma detection for ^11^C-Met was higher (87% versus 49%), slightly more false positives were also reported (3% versus 0%). CRH stimulation before ^18^F-FDG led to somewhat higher diagnostic accuracy compared to ^18^F-FDG without CRH stimulation. Based on single studies, a diagnostic accuracy of 100% was found for ^18^F-FET, ^68^Ga-DOTA-TATE, and ^68^Ga-DOTA-CRH, and was also high in ^68^Ga-pentixafor (86%) and ^13^N-ammonia (90%)—both with one false negative. The exclusion of studies in which histological confirmation was not described (three studies), led to minor changes in results. Diagnostic accuracy was higher for de novo than recurrent pituitary corticotropinomas in mainly ^18^F-FDG (56% versus 35%) and ^68^Ga-pentixafor (100% versus 68%). When MRI was negative or inconclusive, molecular imaging detected a pituitary adenoma in 100% (^68^Ga-DOTA-CRH), 67% (^11^C-Met and ^68^Ga-pentixafor), 22% (^18^F-FDG–PET after CRH stimulation), 17% (^18^F-FDG–PET) and 0 (^13^N-ammonia) patients. For both ^18^F-FDG (± CRH stimulation) and ^11^C-Met, macroadenoma detection was not better than in adenomas sized 7–9 mm (62% versus 82% and 100% versus 100%), while sensitivity for detection of adenomas ≤6 mm was lower (45% and 83%). This was not true for ^68^Ga-DOTA-CRH and ^13^N-ammonia, and the latter even showed lower sensitivity in macroadenoma.

### 3.2. Illustrative Cases

**Figure 1:**
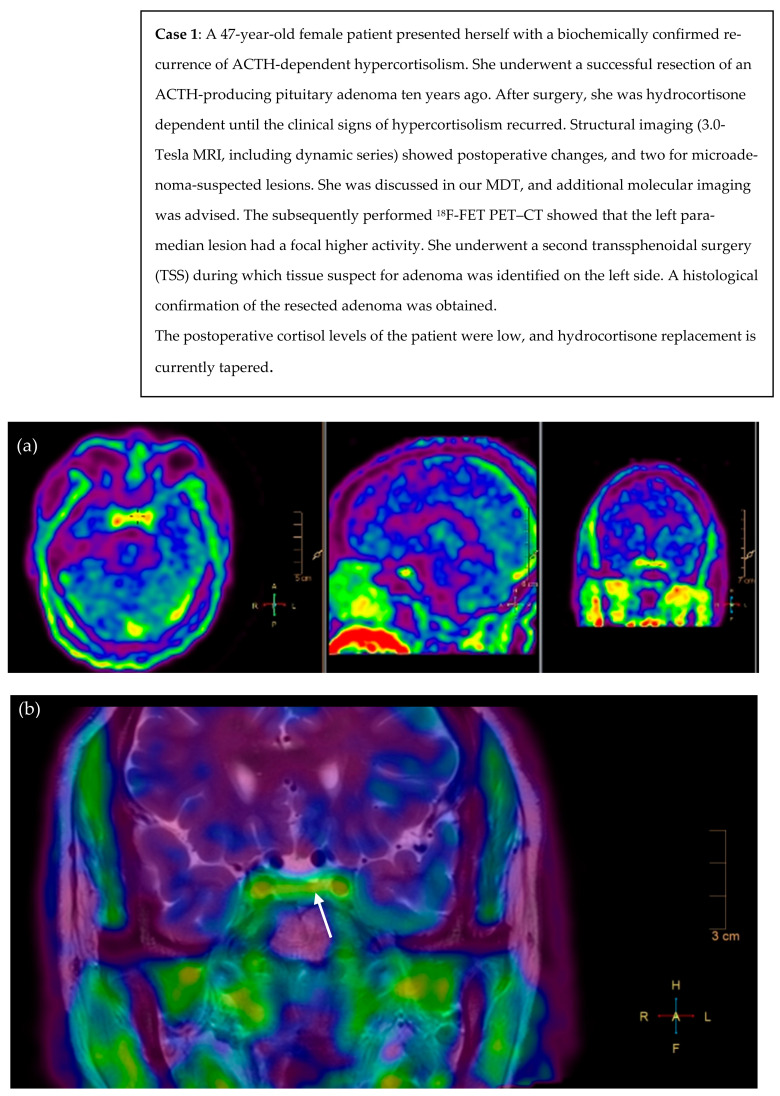
^18^F-FET PET scan and fusion of PET with T2 MR images reveal asymmetrical focal high uptake (in orange) in a pituitary adenoma left paramedian, as shown with the crosshair (**a**) and white arrow (**b**). (**a**) axial, sagittal, and coronal view of PET scan after infusion of 219 MBq ^18^F-FET, (**b**) fusion of PET/MRI (T2-weighted coronal view, pre-gadolinium). L = left, R = right, H = head, F = feet.

**Figure 2:**
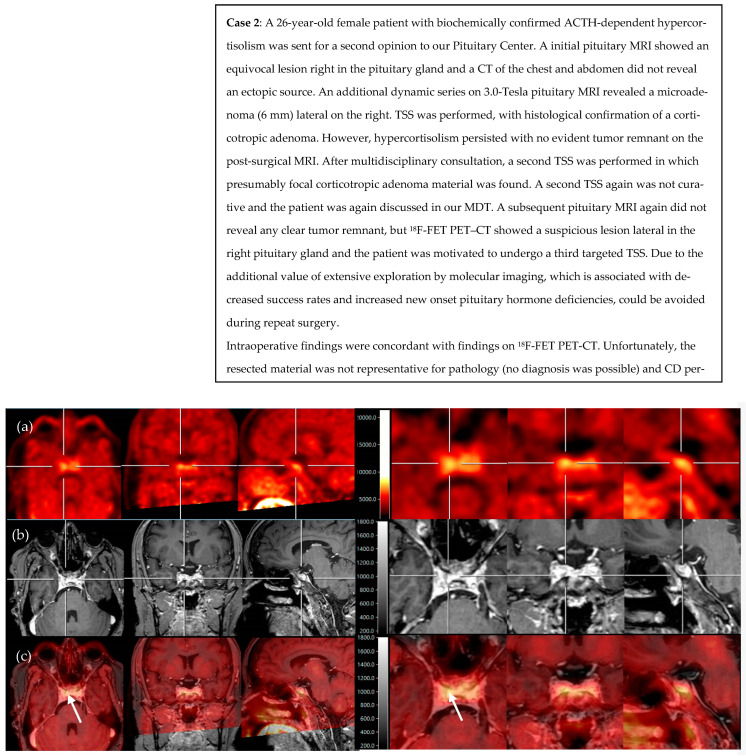
^18^F-FET PET and fusion of PET with post-contrast T1 MR images reveal asymmetrical focal high uptake (in yellow) lateral in the right pituitary, as centered with the crosshair (**a**–**c**) and shown with the white arrows (**c**). (**a**) axial, coronal, and sagittal view of PET scan after infusion of 192 MBq 18F-FET, (**b**) axial, coronal, and sagittal view of 3D T1-weighted, post-gadolinium MRI, and (**c**) axial, coronal, and sagittal view of PET/MRI fusion. Scans on the right side present the same lesion but in more detail.

We present two illustrative CD cases discussed during multidisciplinary counseling in our Pituitary Center, that underwent molecular imaging. Due to pragmatic reasons (reliable production of radioligand and former permission of the Health and Youth Care Inspectorate) and the promising previous results of Berkmann et al., we chose to use ^18^F-FET PET–CT [30]. Case 1 included a patient with CD recurrence and several suspicious lesions on a postoperative pituitary MRI (Figure 1). Case 2 included a patient with persistent CD, with no clear tumor remnant on a postoperative pituitary MRI (Figure 2).

### 3.3. Molecular Imaging in Ectopic Cushing’s Syndrome (See Also Table A1 and Table A2)

A total of 301 patients in 30 articles were described between 2004 and 2022, four of which were prospective (see also Table A1). Tracers included ^68^Ga-SSTR (18 articles, including 8 DOTA-TATE, 6 DOTA-TOC, and 6 DOTA-NOC), ^18^F-FDG (18 articles), ^18^F-DOPA (3 articles), ^68^Ga-DOTA-CRH (1 article), ^68^Ga-pentixafor (1 article), ^11^C-5-HTP (1 article), and ^11^C-Met (1 article). Functional imaging modality was PET (9 articles) and PET–CT (21 articles). We did not include studies describing octreotide/single-photon emission computerized tomography (SPECT) because of their inferior spatial resolution compared to PET. The age ranged from 1 to 80 years, but the vast majority of studies included adult patients with mean ages between 38 and 58 years. Similar to the CD studies, more female than male patients were included, but this difference was less pronounced (1.5:1—146 versus 108; unknown in 47). As expected, most frequently encountered tumors with ectopic ACTH secretion were of pulmonary origin (bronchus carcinoid, pulmonary/small cell lung carcinoma), others included thymic (thymoma/thymic carcinoma), gastro-intestinal (insulinoma, gastrinoma, somatostatinoma, gastric NEC, small bowel carcinoma NET, and carcinoma), pancreatic NET, medullary thyroid carcinoid, olfactory neuroblastoma, and metastatic foci of unknown origin. Rarely encountered underlying tumors were carcinoids of the right atrium, cervix, urinary bladder NET, breast tumor, and paraganglioma/pheochromocytoma.

Diagnostic accuracy: Table A2 shows the diagnostic accuracy for tumor detection in ECS for the different tracers. Tracers that were studied multiple times were ^18^F-FDG, ^68^Ga-SSTR (^68^Ga-DOTA-TATE/-TOC and/-NOC), and ^18^F-DOPA. Among these, ^68^Ga-SSTR showed superior overall sensitivity (59%) compared to ^18^F-FDG (46%) and ^18^F-DOPA (32%). The rate of false positives was high in ^18^F-FDG (23%), while low in ^68^Ga-SSTR (6%) and ^18^F-DOPA (0). The remaining tracers (^68^Ga-pentixafor, ^68^Ga-CRH, ^11^C-Met, and ^11^C-5-HTP) were only studied in very small sample sizes (*n* ≤ 3) but showed very high accuracy. The exclusion of studies that used only PET (and not PET–CT) did not lead to higher accuracy. For both ^18^F-FDG and ^68^Ga-SSTR, sensitivity was higher in recurrent/persistent than de novo CS.

### 3.4. Proposal of Diagnostic Algorithms in ACTH-Dependent Cushing’s Syndrome (See Also Figure 3a,b)

Based on the current literature, we suggest that molecular imaging can be included in the diagnostic algorithm for Cushing’s disease and, therefore, made amendments to the recently proposed algorithms of the consensus statement by Fleseriu et al. [10]. Multidisciplinary counseling remains key, which forms the basis to enable individualization of the diagnostic and treatment approach, and which is also guided by the availability of, and experience with, different diagnostic modalities. In selected cases, we propose the potential to refrain from IPSS and first perform molecular imaging (PET–CT with ^11^C-methionine, ^68^Ga-SSTR, or ^18^F-FET, and if not available, ^18^F-FDG) for de novo CD:

1. If (optimized) structural imaging remains negative or equivocal or shows a microadenoma (<10 mm), and clinical presentation including biochemical testing is suggestive of Cushing’s disease (high “pretest probability”; young women with gradual onset and mildly elevated ACTH levels);
2. If CRH and desmopressin test and whole-body CT (or whole-body ^68^Ga-SSTR PET–CT) in the search for ECS is inconclusive;
3. Presence of contraindications to IPSS (renal failure, blood clotting disorders, or allergy to dye contrast).

**Figure 3:**
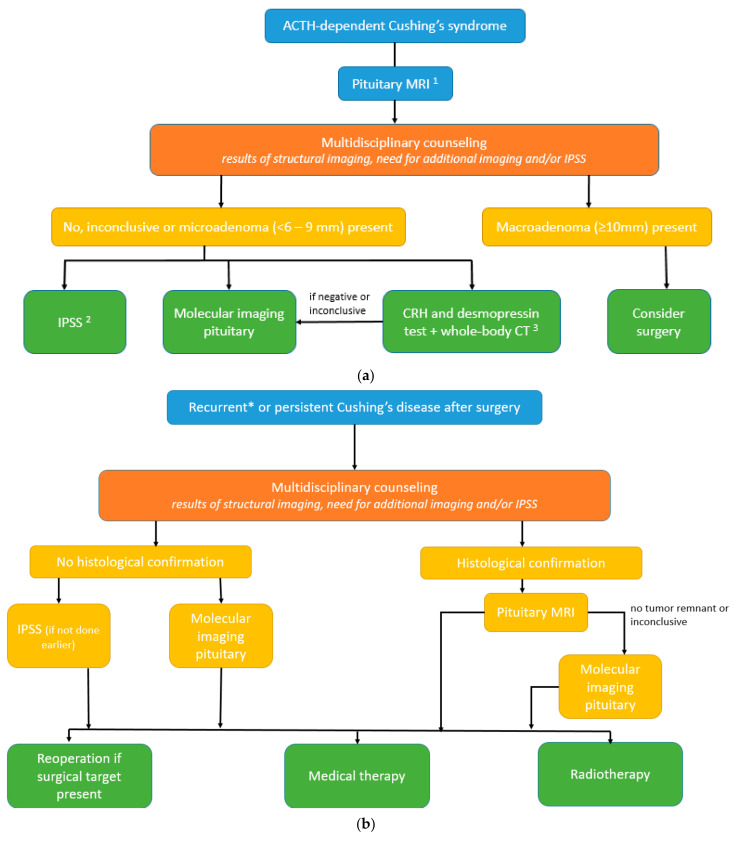
(**a**) Diagnostic algorithm for de novo diagnosis of Cushing’s syndrome.^1^ optimized as proposed by Bashari et al. [17]: Step 1a = conventional MRI: spin echo (SE) pre- and post-gadolinium, if no clear (micro)adenoma >, Step 1b = subsequent MRI sequences: gadolinium-enhanced 3D-spoiled gradient (recalled) echo (3D-SGE/3D-GRE) or gadolinium-enhanced dynamic (dMRI), if still not clear >, Step 2 = subsequent MRI sequences: fluid-attenuated inversion recovery (FLAIR) with gadolinium contrast, constructive interference in steady state (CISS) or isotropic 3D-fast turbo SE and/or use of the ultra-high field (7.0-Tesla) MRI. ^2^ optimally performed with CRH stimulation. ^3^ Alternative: whole-body ^68^Gallium-somatostatin receptor PET–CT. (**b**) Algorithm for persistent or recurrent Cushing’s disease. * In selected cases of Cushing’s disease with the equivocal biochemical response after surgery.

Next to the results of structural imaging and the need for additional imaging or IPSS, other relevant aspects such as age, child wish, and remaining pituitary function (hypopituitarism) are also discussed during multidisciplinary counseling in persistent or recurrent CD. We propose the potential to perform functional imaging (PET–CT with ^11^C-methionine, ^68^Ga-SSTR, or ^18^F-FET, and if not available, ^18^F-FDG) in the following cases:

1. Persistent or selected cases of recurrent Cushing’s disease (equivocal biochemical response) after transsphenoidal surgery (TSS) and without histological confirmation.
2. Persistent or selected cases of recurrent Cushing’s disease (equivocal biochemical response) after TSS and with histological confirmation, but no or inconclusive adenoma remnant localization on the pituitary MRI (illustrative cases 1 and 2).

## 4. Discussion

The definitive diagnosis of Cushing’s disease in the presence of pituitary microadenoma remains a continuous challenge in individual cases since most corticotroph adenomas are microadenomas. While the diagnostic accuracy of structural imaging in the detection of these microadenomas has improved over the last decades, the diagnostic accuracy to detect all microadenomas is still limited. On the other hand, improved sensitivity of structural imaging may lead to the detection of incidentalomas (false positives) [9,25]. This structured review clearly supports the added value of molecular imaging—co-registered or combined with structural MRI—in the diagnostic work-up of ACTH-dependent CS in selected cases. In de novo patients, we propose potentially refraining from IPSS and performing molecular imaging first (PET–CT with ^11^C-methionine, ^68^Ga-SSTR, or ^18^F-FET, and if not available, ^18^F-FDG) in the following cases: (1) if (optimized) structural imaging remains negative or equivocal or shows a microadenoma (<10 mm) and clinical presentation including biochemical testing is suggestive of Cushing’s disease (high “pretest probability”), (2) if CRH and desmopressin whole-body CT, in search for ECS, is inconclusive, or (3) presence of contraindications to IPSS (renal failure, blood clotting disorder, or allergy to dye contrast). In persistent or recurrent CD, we propose the possibility of performing molecular imaging in the following cases: (1) persistent or selected cases of recurrent Cushing’s disease (equivocal biochemical response) after TSS and without histological confirmation, before the use of IPSS (if not performed previously) or (2) in persistent or selected cases of recurrent Cushing’s disease (equivocal biochemical response) after TSS and with histological confirmation, but with equivocal tumor remnant localization on structural imaging (illustrative cases).

At present, the international consensus guideline allows for different diagnostic modalities after the biochemical confirmation of ACTH-dependent CS [10]. This allows for increased tailor-made diagnostic strategies, depending on, among others, the institutional availability and experience with both noninvasive and invasive diagnostic tests and modalities. Several alternative noninvasive diagnostic strategies after the optimization of pituitary MRI protocols, but before the use of IPSS, have been described. Isidori et al. found that a combination of dynamic testing, using both the CRH test and dexamethasone suppression test, led to a sensitivity of 97% and specificity of 94% in the correct distinguishing of CD from an ectopic source if both tests are positive [33]. In the published literature up to date, this was not confirmed for the high dose dexamethasone suppression test (which exists in multiple variants), as recently reviewed by Ferriere and Tabarin [34], while other studies indeed show high diagnostic accuracy of CRH testing in the differentiation between CD and ECS, so the usefulness of the CRH test and the high dose dexamethasone suppression test remains controversial. Another alternative noninvasive strategy was proposed by Frete et al., using CRH and desmopressin tests in combination with pituitary MRI and thin-slice whole-body CT. They found very high diagnostic accuracy when both tests and imaging were conclusive (e.g., CD: negative pituitary MRI and CT in combination with a positive CRH and desmopressin test, ECS: negative pituitary MRI and negative CT and a negative CRH and desmopressin test) and calculated that (recommendation of) IPSS could be omitted in about half of the patients [35]. The latter alternative strategy has now been incorporated in the update of the international clinical guidelines and states that if both tests are positive and no focus on the whole-body CT scan is found, CD can be assumed, while the opposite accounts for ECS, especially in the setting of a high pretest probability [10]. It should be mentioned that expert opinions differed on this last proposal and it warrants further investigation. Above that, these alternative diagnostic approaches also have their shortcomings. As mentioned before, the availability of CRH is decreasing (and the alternative desmopressin requires further research) and tests may also be discordant, again leading to the need for invasive IPSS.

It should be noted that although the diagnostic accuracy of IPSS to distinguish CD from ECS is very high, IPSS also gives false negatives (approximately 10–15%) and false positive results, leading to an estimated specificity between 90 and 95% [36,37]. Given that IPSS also is an invasive procedure with associated risks, only reliable in highly experienced hands, and lacks correct pituitary adenoma lateralization, there is a persistent unmet need to improve the stepwise (noninvasive) diagnostic approach in ACTH-dependent CS [37,38].

Part of this need is fulfilled by optimization of the dedicated pituitary MRI (thin slice, small field of view, dynamic contrast acquisition) and using higher magnetic field strengths (3.0 and even 7.0 Tesla), leading to increased detection of microadenomas. However, in this structured review, we found that MRI, even in those studies that included the more sensitive additional SPGR sequence, still failed to clearly detect a pituitary adenoma in 24 to 28% of CD patients. Molecular imaging, combined with CT or MRI, or co-registered with MRI, combines both anatomical and functional tissue information and appears to provide added value also for the imaging of pituitary adenomas. Large cohorts as well as individual small case series, report on the amount of (incidental) pituitary uptake of radioligands and their clinical significance, in both the general population and in different pituitary conditions such as (functioning) adenomas or carcinomas [22,39,40,41,42,43,44]. More specifically, the number of original studies on molecular imaging modalities in the detection of CD and ECS is increasing. While molecular imaging is already studied and used in ECS detection for a longer period, most studies on the clinical use of CD are from the last years. One of the advantages of molecular imaging is that detection of adenomas seems less reliant on tumor size, which is particularly relevant for corticotropinomas with their predominantly small sizes [23,24]. In this structured review, we also found that detection rates did not decrease notably in adenomas sized ≤ 6 mm in comparison to larger adenomas. Approximately half and 87% of pituitary adenomas were detected using ^18^F-FDG and ^11^C-Met PET(–CT), respectively. PET–CT using ^18^F-FET and three ^68^Ga-labeled radioligands even showed higher sensitivity (up to 100%), but were only studied in smaller samples: *n* = 9 for ^18^F-FET, *n* = 7, 7, and 24 for ^68^Ga-DOTA-TATE, -pentixafor and –DOTA-CRH, respectively. This should be taken into consideration when choosing an appropriate radioligand for molecular imaging in the detection and localization of pituitary microadenoma. Not many false positives were reported. With regard to the ^18^F-FDG studies included in this review, most authors concluded that its diagnostic use in CD is mainly complementary since some extra cases were detected on PET–CT that were not (clearly) seen on conventional MRI [20,22,23]. Stimulation with CRH can lead to increased ^18^F-FDG uptake, possibly leading to higher detection rates of pituitary adenoma in CD [24]. In studies that reported on both ^18^F-FDG and ^11^C-Met, diagnostic accuracy was higher in ^11^C-Met [21,26]. Four studies reported superior adenoma detection and localization using ^11^C-Met compared to other (structural) imaging techniques, while in another study, this appeared not true when using the (additional) SPGR MRI sequence [19,21,25,26,30]. Given the high predictive value for adenoma detection and localization, multiple studies proposed a useful role of ^11^C-Met PET–CT in treatment planning and/or in recurrent/residual cases to distinguish postoperative changes from adenoma tissue; a distinction less easily made by MRI [19,25,30]. This also accounts for ^68^Ga-DOTA-TATE and ^68^Ga-DOTA-CRH [29,31]. The specifically developed ^68^Ga-DOTA-CRH and ^68^Ga-pentixafor for the detection and localization of corticotroph adenoma showed very promising results, and sensitivity was higher than optimized MRI (SPGR/dynamic) with 100% and 86% sensitivity, respectively [29,32]. Overall, several authors suggested that molecular imaging with PET–CT could be a complementary diagnostic tool to MRI and/or IPSS in Cushing’s adenoma, especially when not available or inconclusive, in difficult cases, or when IPSS can even be omitted [20,21,25,26].

Besides these promising results, molecular imaging also has some limitations and disadvantages compared to IPSS. For instance, the sensitivity of molecular imaging in the diagnosis of CD ranged between 49% (^18^F-FDG) and 100% (^68^Ga-DOTA-TATE, ^68^Ga-DOTA-CRH, and ^18^F-FET, see also Table 2), which is lower than the sensitivity range reported for IPSS (80–100%) [36,37]. Concerning specificity, false positive cases are very rare in IPSS and to date have only been reported in two patients using molecular imaging (^11^C-Met). Theoretically, other sellar lesions, such as (non-)functioning pituitary adenoma, can lead to false positives when using molecular imaging for the diagnosis of CD in ACTH-dependent hypercortisolism, since the uptake of ^18^F-FDG and ^11^C-Met has also been described in these lesions [19,39]. In addition, the interpretation of molecular imaging requires specific expertise that, as is the case for any new technique, is subject to standardization, optimization, and a learning curve. Consequently, outcomes are still reviewer dependent. This can lead to interrater variability, as shown by the study of Boyle et al., in which 4/27 (^18^F-FDG hrPET without CRH stimulation) and 1/27 (^18^F-FDG hrPET with CRH stimulation) corticotropinoma were reviewed as such by only one of two neuroradiologists [24]. As mentioned before, accessibility to molecular imaging is currently limited to some specialized referral centers, and some isotopes, such as ^11^C-Met, require the availability of an expensive cyclotron, and, as stated above, experienced nuclear radiologists and validation for correct interpretation are required per (expert) center. Therefore, we conclude that molecular imaging is still complementary, serving as part of the whole in the diagnostic work-up of ACTH-dependent CS.

For the performance of additional (molecular) imaging, such as in the case of IPSS, patients need to be referred to expert centers with demonstrable expertise in this specific technique. In ACTH-dependent CS, but also for rare diseases in general, it is, therefore, of the utmost importance to share knowledge about and availability of diagnostic opportunities, which is (made) possible in a network context. With the establishment of such networks, complex cases can be discussed within a broad team of (multidisciplinary) experts and physicians who can clearly inform their patients about possibilities for further diagnostic or treatment options after extensive and optimal counseling, including shared decision making.

The diagnostic use of molecular imaging has been studied more extensively and for a longer period in the case of neuroendocrine tumors with ECS. The tumors are also rare and represent a heterogeneous group of patients in which tumors can occur throughout the whole body, but are mostly found in the chest or abdomen. Former studies already advocated a more prominent role of molecular imaging in diagnostic algorithms [45,46,47]. In the present structured review, we found sensitivity rates of 46%, 59%, and 32% for ^18^F-FDG, ^68^Ga-SSTR, and ^18^F-DOPA PET(-CT), respectively. Most authors of included ECS studies on ^18^F-FDG PET(–CT), concluded that it should be used as a complementary diagnostic tool since detection rates were not better than (less costly) conventional imaging modalities (CT or MRI) [45,48,49,50,51]. However, in the case of negative conventional imaging, or to distinguish true from false positive lesions in inconclusive scans, it can be very helpful [49,52,53,54,55]. Two studies highlighted the dependency of ^18^F-FDG on metabolic activity, such as tumor proliferation, with aggressive and invasive tumors being better visualized than tumors with low metabolic activity [47,50]. ^18^F-FDG was also found superior to ^68^Ga-SSTR imaging in suspected metastasis or in the differentiation between pulmonary infections and ACTH-secreting bronchial tumors in found lung nodules [56,57], while ^68^Ga-SSTR appeared superior to ^18^F-FDG for de novo ECS tumor detection [51,57]. Conclusions of authors of studies on ^68^Ga-SSTR imaging in the tumor detection of ECS were not concordant: while some studies reported high diagnostic accuracy [51,58,59,60] and found its use helpful in tumor staging and treatment decision making [59,60,61] or even limiting the need for invasive diagnostic procedures [58], others stated that its use should be complementary when conventional imaging is negative or to enhance the positive predictive value of previously found lesions [31,55,62,63,64]. Dutta et al. reported that ^68^Ga-DOTA-TOC PET–CT was not useful in the detection of thymic carcinoid, and Varlamov et al. suggested that previously reported results on ^68^Ga-SSTR imaging for tumor detection in ECS are probably subject to publication bias [65,66]. Overall, we conclude that molecular imaging, especially ^68^Ga-SSTR PET–CT, can be of additional value in the diagnostic work-up for ECS.

Recently, three reviews highlighted the potential use of new emerging imaging techniques in Cushing’s syndrome [17,18,67]. The current paper, however, is the first that structurally reviews all of the available literature, providing an up-to-date synergistic overview, addressing multiple aspects of the diagnostic work-up of ACTH-dependent CS, and proposing amendments to the diagnostic algorithms of the current consensus statement. Since most studies were retrospective and included a limited number of patients, we propose future research to confirm results in a prospective study including a larger cohort, ideally also comparing diagnostic accuracy to the results of IPSS. This is in line with one of the recommendations of the current consensus statement on the diagnosis of CD, which suggests that combining structural with molecular imaging will likely improve diagnostic work-up, but more data on the clinical use of molecular imaging are needed [10].

## 5. Conclusions

Altogether, with the upcoming availability of highly sensitive and discriminative imaging modalities in the detection of pituitary microadenoma in ACTH-dependent CS, the potential to refrain from IPSS is rising. This seems justified for selected cases, including even microadenomas smaller than 6 mm, in which the MDT agrees on the high pretest probability of Cushing’s disease and provided that the center has validated and demonstrated expertise with specific techniques. Centers differ in their diagnostic capabilities and work-up strategies, which makes good communication and sharing knowledge within a Cushing’s network key for this challenging disease.

## Figures and Tables

**Table 2:** Diagnostic accuracy of molecular imaging in Cushing’s disease.

		^18^F-FDG (8 Articles)	^18^F-FDG + CRH (1 Article)	^18^F-FET (1 Article)	^11^C-Met (5 Articles)	^68^Ga-DOTA-TATE (1 Article)	^68^Ga-Pentixafor (1 Article)	^68^Ga-DOTA-CRH (1 Article)	^13^N-Ammonia (1 Article)
All	True +	49% (47/96)	56% (15/27)	100% (9/9)	87% (53/61)	100% (7/7)	86% (6/7)	100% (24/24)	90% (9/10)
	False −	51% (49/96)	44% (12/27)	0% (0/9)	10% (6/61)	0% (0/7)	14% (1/7)	0% (0/24)	10% (1/10)
	False +	0% (0/96)	0% (0/27)	0% (0/9)	3% (2/61)	0% (0/7)	0% (0/7)	0% (0/24)	0% (0/10)
Histological confirmation	True +	52% (45/87)	56% (15/27)	100% (9/9)	85% (45/53)	100% (7/7)		100% (24/24)	90% (9/10)
	False -	48% (42/87)	44% (12/27)	0% (0/9)	11% (6/53)	0% (0/7)		0% (0/24)	10% (1/10)
	False +	0% (0/87)	0% (0/27)	0% (0/9)	4% (2/53)	0% (0/7)		0% (0/24)	0% (0/10)
De novo	True +	56% (44/79)	61% (14/23)		88% (28/32)		100% (4/4)	100% (24/24)	88% (7/8)
	False -	44% (35/79)	39% (9/23)		10% (3/32)		0% (0/4)	0% (0/24)	13% (1/8)
	False +	0% (0/79)	0% (0/23)		3% (1/32)		0% (0/4)	0% (0/24)	0% (0/8)
Recurrent	True +	35% (6/17)	50% (2/4)		85% (17/20)	100% (7/7)	68% (2/3)		100% (2/2)
	False -	65 (11/17)	50% (2/4)		15% (3/20)	0% (0/7)	33% (1/3)		0% (0/2)
	False +	0% (0/17)	0% (0/4)		0% (0/32)	0% (0/7)	0% (0/3)		0% (0/2)
MRI negative/inconclusive	True +	17% (4/23)	22% (2/9)		67% (8/12)		67% (2/3)	100% (4/4)	0% (0/1)
	False -	83% (19/23)	7/9 (78%)		25% (3/12)		33% (1/3)	0% (0/4)	100% (1/1)
	False +	0% (0/23)	0% (0/9)		8% (1/12)		0% (0/3)	0% (0/4)	0% (0/1)
Micro-adenoma (unspecified)	True +	75% (9/12)		100% (9/9)	94% (16/17)		86% (6/7)		
	False -	25% (3/12)		0 (0/9)	0% (0/17)		14% (1/7)		
	False +	0% (0/12)		0 (0/9)	6% (1/17)		0% (0/7)		
≤6 mm	True +	45% (10/22)	57% (4/7)		83% (5/6)			100% (10/10)	100% (3/3)
	False -	55% (12/22)	43% (3/7)		0% (0/6)			0% (0/10)	0% (0/3)
	False +	0% (0/22)	0% (0/9)		17% (1/6)			0% (0/10)	0% (0/3)
7–9 mm	True +	82% (9/11)	100% (3/3)		100% (5/5)			100% (7/7)	100% (2/2)
	False -	18% (2/11)	0% (0/3)		0% (0/5)			0% (0/7)	0% (0/2)
	False +	0% (0/11)	0% (0/3)		0% (0/5)			0% (0/7)	0% (0/2)
≥10 mm	True +	62% (16/26)	88% (7/8)		100% (7/7)			100% (7/7)	80% (4/5)
	False -	38% (10/26)	13% (1/8)		0% (0/7)			0% (0/7)	20% (1/5)
	False +	0% (0/26)	0% (0/8)		0% (0/7)			0% (0/7)	0% (0/5)

^11^C-Met = ^11^C-Methionine; ^18^F-FDG = ^18^F-Fluorodeoxyglucose; ^68^Ga = ^68^Gallium; CRH = corticotropin-releasing hormone; ^18^F-FET = ^18^F-fluoroethyl-L-tyrosine; given as % (total positive patients/total tested patients).

## Data Availability

Not applicable.

## Appendix A

**Table A1:** Original studies on functional imaging in ectopic Cushing’s syndrome.

Ref.	Author	Year	Tracer	Imaging Modalities	Population			Aim	Results		Conclusions of Authors
					*N*	Age	Sex *		Diagnosis	Imaging	
[48]	Pacak et al. (National Institutes of Health, USA) **Prospective**	2004	^18^F-FDG (20 mCi)	PET	17	43 ± 13 (22–69)	59%	Assess ^18^F-FDG–PETsensitivity for detection of ACTH-secreting tumors and comparison with conventional imaging.	(Pulmonary) carcinoid, insulinoma, gastrinoma, somatostatinoma, olfactory esthesioneuroblastoma, SCLC, and metastatic carcinoid	Population: ACTH-secreting tumors Routine imaging + FDG–PET, H-OCT if L-OCT was negative > localized in 13/17 (76%), occult in 4/17 (24%) ECS detection: true positives/false positives - CT (*n* = 17): 9/17 (53%)/3/17 - MRI (*n* = 16): 6/16 (37%)/3/16 - ^18^F-FDG–PET (*n* = 17): 6/17 (35%)/4/17 Localization: ^18^F-FDG -PET concordant with CT and/or MRI in 6 true positives; L-OCT in 8 true positives. ^18^F-FDG–PET did not reveal additional tumors beyond those identified on CT or MRI; the smallest detected lesion = 1.3 cm. - positive scans were typically more aggressive (NET, olfactory neuroblastoma, SLCL) - negative scans were less aggressive (pulmonary carcinoids) or a form of tumoral hibernation Outcome: 12 underwent surgery: ACTH staining: 8+, 1- but hypercortisolism resolved after resection, 2 not available 2 with + imaging and biopsy-proven ACTH-secreting tumors did not undergo surgical resection 4 remained with occult disease	^18^F-FDG–PET is inferior to CT/MRI and does not detect additional ACTH-secreting tumors causing CS (occult tumors on CT/MRI). Because hyperplastic adrenal glands may show ^18^F-FDG–PET and/or OCT uptake, an adrenal ACTH-secreting lesion may be obscured. Recommend that CT, MRI, and L-OCT be used to screen for tumors. Modalities are complementary: single + study may represent false +, more than 1 + study = confirm true lesion.
[68]	Zemskova et al. (National Institutes of Health, USA) **Prospective**	2010	^18^F-FDG + F-DOPA	PET	11 (41 total)	Proven: 45 ± 13.6 (23–69), occult: 54 ± 14.4 (33–82) (total)	Proven: 43%, occult: 73% (total)	Evaluate the utility of ^18^F-FDG and other modalities.		Population: 41 patients with ECS based on IPSS > 11/41 (27%) remained occult, and 30/41 (73%) resected tumors. In tumor-identified patients (n = 30): sensitivities per patient/positive predictive value per lesion/proportion of FP lesions/fraction of patients with ≥ 1 false+ findings - CT—neck/chest/abdomen/pelvis (*n* = 30) = 28/30 (93%)/66%/34%/50% - MRI—neck/chest/abdomen/pelvis (3T, *n* = 29) = 26/29 (90%)/74%/26%/31% - ^18^F-FDG–PET (*n* = 11) = 7/11 (64%)/53%/47%/18% - F-DOPA-PET (*n* = 11) = 6/11 (55%)/100%/0%/x In all patients with EAS (n = 41): sensitivities per patient/positive predictive value per lesion/proportion of FP lesions - CT—neck/chest/abdomen/pelvis = 28/41 (68%)/57%/43% - MRI—neck/chest/abdomen/pelvis (3T) = 26/40 (65%)/67%/ 33% - ^18^F-FDG–PET = 7/14 (50%)/50%/50% - F-DOPA-PET = 6/13 (46%)/89%/11% PET detected only lesions also seen by CT/MRI; abnormal F-DOPA-PET improved PPV of CT/MRI.	High sensitivity and positive predictive value suggest thoracic CT/MRI + L-OCT for initial imaging, with lesions confirmation by 2 modalities.
[53]	Kumar et al. (Guy’s Hospital, London)	2006	^18^F-FDG	PET	3	44 + 24 + + 74	33%	Report on the use of ^18^F-FDG–PETscanning in the evaluation of ECS.	Carcinoid: right atrium and lung (2)	ECS localization: - BIPSS (*n* = 2): ECS in 2/2 - X-thorax (*n* = 1), - in 1/1 - CT—chest (*n* = 3): + in 1/3 (nodule lung), − in 2/3 - CT—abdomen (*n* = 2): + but bilateral adrenal hyperplasia - MRI—chest (*n* = 2): + in 1/2, − in 1/2 - MRI—pituitary (*n* = 2): − in 2/2 - ^18^F-FDG–PET whole body (*n* = 3): + in 3/3	^18^F-FDG–PET assisted in localizing small metabolically active NETs, suggesting this imaging modality may have a useful role in identifying NET-causing CS as a result of ECS. ^18^F-FDG–PET is useful where conventional imaging modalities fail/changes of uncertain significance.
[54]	Moraes et al. (Hospital Universitário Clementino Fraga Filho, Brazil)	2008	^18^F-FDG	PET	2	31 + 53	50%	Report utility of ^18^F-FDG–PETin localization of 2 ECS tumors, where conventional imaging failed to definitively identify lesions.	Carcinoid + possible cervix	ECS localization: - BIPSS (*n* = 1): ECS in 1/1 - CT—thorax (*n* = 2): + in 1/2 (lung nodule), − in 1/2 - MRI—abdomen/pelvis (*n* = 1): + in 1/1 (hepatic, iliac, femoral, and lumbar secondary implants) - MRI—pituitary (*n* = 2): − in 2/2 - ^18^F-FDG–PET (*n* = 2): + in 2/2	C/^18^F-FDG–PETmay have a role in the investigation of an ECS where conventional imaging studies were not diagnostic.
[49]	Xu et al. (Rui Jin Hospital, China)	2009	^18^F-FDG (0.12–0.15 mCi/kg)	PET–CT	5	50 ± 14 (27–64)	60%	Report on the use of ^18^F-FDG PET–CT in localization of EAS tumors in ECS.	Pulmonary carcinoma (2), thymoma (1), thymic carcinoma (1)	ECS localization: - BIPSS (*n* = 2): ECS in 2/2 - MRI—pituitary (*n* = 4): − in 3/4, + in 1/4 (possible microadenoma) - CT—abdomen (*n* = 5): − in 1/5, + in 4/5 (bilateral adrenal hyperplasia) ^18^F-FDG PET–CT: + in 5/5 positive 5 NETs and also 1 pneumonia, 2 hyperplasia adrenals Outcome: 4/5 resection: 3 biochemical releases, 1 clinical release 1/5 died due to severe infection and electrolyte disorders	^18^F-FDG PET–CT increases the accuracy of tumor localization and further improves prognosis by curative resection. CT may localize the source better and PET reduces the false + rate. PET provides metabolic information and may reveal lesions that potentially mislead CT.
[50]	Doi et al. (Tokyo Medical and Dental University Graduate School, Japan)	2010	^18^F-FDG	PET	9 (16 total)	58.4 ± 19 (total)	44% (total)	Evaluate the clinical, endocrinological, and imaging features, management, and prognosis of 16 EAS.	Lung carcinoma (3), thymic hyperplasia (1), bronchus carcinoma (1), olfactory neuroblastoma (1), gastric NEC (1), pancreatic NEC (1)	Population: 16 EAS: 10/16 proven and 6/10 occult/unknown ECS detection: - IPSS: 13/15 (87%) no ACTH gradient - CT/MRI: + in 8/16 (50%) and − in 8/16 (50%) - ^18^F-FDG–PET: + in 1/9(11%, SCLC, also + on CT/MRI/SRS), - in 8/9 (1 + on CT/MRI, 2 + on SRS, 5 also - other)	To localize ECS, a combination of dynamic endocrine tests and imaging tests, including anatomical modalities (CT and MRI) and functional modalities (SRS and ^18^F-FDG–PET) is required. ^18^F-FDG–PETis known to identify tumors with high proliferative activities: modality seems limited to localizing malignant tumors with a highly aggressive and invasive nature.
[66]	Dutta et al. (All India Institute of Medical Sciences, India)	2010	^68^Ga-DOTA-TOC	PET–CT	2	29 + 23	50%	Describe the utility of ^68^Ga-DOTA-TOC PET–CT and clinic-pathological features in 4 thymic carcinoid tumors.	Thymic carcinoid tumors (4)	Population: 2 cases of thymic carcinoid (stage 2 and 3): ECS localization: - BIPSS: ECS in 2/2 - CECT—chest: + in 2/2 - MRI—pituitary: − in 2/2 - ^68^Ga-DOTA-TOC PET–CT: − in 2/2	Unlike pulmonary and abdominal carcinoids, ^68^Ga-DOTA-TOC PET–CT has not proven useful in thymic carcinoids.
[45]	Ejaz et al. (University of Texas MD Anderson Cancer Center)	2011	^18^F-FDG	PET	6 (20 total)	48 [19–75] (total)	40% (total)	Study our institutional experience with CS-EAS and further understand this entity in a cancer center.	Bronchus carcinoid (9), SCLC (9), medullary thyroid carcinoid (4), pancreatic NET (3), thymic carcinoma (3), urinary bladder NET (1), small bowel carcinoma (1), metastatic NET (1)	ECS localization: - BIPSS (*n* = 8): EAS in 8/8 - pituitary MRI (*n* = 34): − in 31/34 (91%), + in 3/31 (9%) (incidental pituitary abnormalities) - CT/MRI—chest (*n* = 37): + in 25/37 (68%) - CT/MRI—abdomen (*n* = 32): + in 9/32 (28%) - ^18^F-FDG–PET(*n* = 6): + in 4/6 (67%; all also on CT/MRI), - in 2/6 (33%)	^18^F-FDG–PET localized ACTH sources in four of six patients in whom primary tumor was also observed on less expensive cross-sectional imaging studies.
[47]	Boddaert et al. (Georges Pompidou European Hospital, France)	2012	^18^F-FDG + ^18^F-DOPA	PET	6 (12 total)	40 (16–63) (total)	57% (total)	Revisit characteristics and outcomes of ACTH-secreting bronchial carcinoid tumor responsible for CS.	Bronchial carcinoids tumors (14; of which 11 are typical and 3 atypical)	Population: 14 bronchial carcinoid tumors causing CS. ECS localization: - BIPSS (*n* = 6): ECS in 6/6 - Pituitary MRI (*n* = 14): − in 10/14 (71%), + in 4/14 (29%; 3/4 suggestive adenoma, 1/4 Rathke’s cleft cyst) - X-thorax (*n* = 14): − in 12/14 (86%), + in 2/14 (14%; 1 basithoracic nodule + 1 diffuse reticulonodular lesion) - CT—thorax *(n* = 14): + in 9/14 (64%), − in 5/14 (36%) + in other 5: tumor detected with mean delay 68 months - CT—abdominal (*n* = 12): + in 4/12 (33%; bilateral adrenal hyperplasia and 2/12 abnormal adrenal nodular pattern) - ^18^FDG-PET: + in 3/4 (75%, moderately abnormal), − in 1/4 - ^18^F-DOPA-PET: + in 1/2 (50%), − in 1/2 (50%)	^18^F-FDG–PETis of limited use in these tumors because of its low metabolic activity. New and more specific markers of carcinoid tumors are currently available, including ^18^F-DOPA or ^11^C-5 HTP.
[58]	Giraldi et al. (European Institute of Oncology, Milan, Italy)	2013	^68^Ga-DOTA-TOC (3 MBq/kg)	PET–CT	5	37–67	20%	Report experience with ^68^Ga-DOTA-TOC PET–CT in 5 patients with clinical and biochemical evidence of EAS tumors of unknown origin.	Typical carcinoid (3)	ECS localization: - MRI—pituitary: − in 3/5, + in 2/5 (pituitary adenoma) - BIPSS (*n* = 2): ECS in 2/2 - Conventional imaging (*n* = 5): + in 2/5 (40%), − in 3/5 (60%) - ^68^Ga-DOTA-TOC PET–CT: + in 5/5 (100%), − in 0/5 including 1 false + (left adrenal: hyperplasia)	^68^Ga-DOTA-TOC PET–CT can direct diagnosis in a noninvasive way, eliminating the need for an invasive procedure. However, physiological uptake in the pituitary/spleen/adrenals/head/pancreas may limit sensitivity (false + in one patient).
[62]	Özkan et al. (Instanbul University Instanbul Medical Faculty, Turkey)	2013	^68^Ga-DOTA-TATE (3–4 mCi) + ^111^In (5 mCi)	PET–CT + SPECT–CT	5 (19 total)	37.8	32%	Evaluate the value of SSTR imaging with OCT and ^68^Ga-DOTA-TATE in localizing ectopic ACTH-producing tumors.	Pulmonary carcinoid (6), pancreatic NET (1), metastatic foci atypical carcinoid of unknown origin (1)	Population: 8/19 (42%) ectopic site detected ECS localization: Of ectopic foci: + in 7/8 (88%) on OCT or ^68^Ga-DOTA-TATE PET–CT, − in 1/8 (metastatic foci) 6 pulmonary carcinoids: - 4/6 + on OCT (all also on CT) - 1/6 - on OCT, follow-up CT after 3 years: nodule upper lobe (carcinoid) - 1/6 + on ^68^Ga-DOTA-TATE PET–CT, prior false- evaluated on CT (atypical pulmonary carcinoid) 1 pancreatic NET: detected on MRI + OCT scan 1 metastatic focus atypical carcinoid after resection mediastinal mass + lymph nodes: metastatic lymph nodes and bone lesions on OCT; ^68^Ga-DOTA-TATE PET–CT showed progression of the disease. OCT: performed in 16/19 ^68^Ga-DOTA-TATE PET–CT: performed in 5/19 11 patients: EAS site could not be detected; - 10/11 - (scans + uptake) - 1/11: ^68^Ga-DOTA-TATE PET–CT false + (mass adrenal gland on CT with moderate 68Ga uptake > adenoma without ACTH staining)	Somatostatin receptor imaging has a complementary role with radiological imaging in localizing ectopic ACTH secretion sites. PET–CT imaging with ^68^Ga peptide conjugates is a promising new modality for this indication. Since pulmonary carcinoid tumors are responsible for ECS in most cases, thoracic regions must be evaluated with great care.
[69]	Wahlberg and Ekman (Linköping University, Sweden)	2013	^11^C-5-HTP	PET	1	63	0%	Describe diagnostic challenges to find a tumor in CS secondary to ECS in 2 cases with (a)typical pulmonary carcinoid.	Pulmonary carcinoid (atypical)	ECS localization: - BIPSS: ECS in 1/1 - MRI—pituitary: - in 1/1 - CT—neck/thorax/abdomen: enlargement of the left adrenal - ^11^C-5 HTP–PET: + in 1/1: 8 mm mass left lung with focal uptake in retrospect: the same shadow was present on thoracic CT, previously assessed as a vessel	Diagnostic evaluation time is limited due to the aggressive course in ECS. We suggest that ^11^C-5-HTP–PET could be considered early as a secondary diagnostic tool when primary CT and/or MRI fail to show tumor.
[52]	Gabriel et al. (La Timone & North University Hospital, France) **Prospective**	2013	^68^Ga-DOTA-TATE + ^111^In	PET–CT + SPECT	5 (32 total)	22–80 (total)	41% (total)	Perform head-to-head comparison between ^68^Ga-DOTA-TATE PET–CT and standard imaging work-up of NET that included multiphasic CT, liver MRI, and SRS SPECT.	Lung NET (4; of which 2 typical and 2 atypical)	Population: 32 NET > 5 cases ectopic ACTH secretion: 4 lung NETs and 1 remained occult after all imaging ECS localization: - Conventional imaging (CT/MRI): + in 2/5 (40%), - in 3/5 (60% of which 1 typical, 1 atypical, and 1 occult) - ^68^Ga-DOTA-TATE PET–CT: + in 2/5 (40%; both - on CT), − in 3/5 (60%; 2 false- and 1 occult also on CT)	^68^Ga-DOTA-TATE PET–CT detected a similar number of sites with a combination of SRS, liver MRI, and thoraco-abdominopelvic CT on region-based analyses and missed half of the primary lung carcinoids with ECS.
[55]	Kakade et al. (KEM Hospital Mumbai, India)	2013	^68^Ga-DOTA-TATE+ ^18^F-FDG	PET + CT + MRI (3D VIBE)	6 (17 total)	42.67 (20–63)	67%	Analyze clinical, biochemical, and imaging characteristics; management strategies and outcomes of EAS.	Bronchial carcinoids (6), thymic (1), metastatic (2), medullary thyroid carcinoid (1)	ECS localization: - IPSS: ECS in 8/8 - MRI—pituitary: − in 12/17 (71%), + in 5/17 (29%; suspicious microadenoma > TSS > uncured) - CT (*n* = 17): + in 15/17 (88%), − in 2/17 (both + on ^68^Ga- DOTA-TATE PET–CT) - MRI (3D VIBE): true+ (+MRI in CD) = 111, false- (-MRI in CD) = 90, false+ (+MRI in EAS) = 5, false- (-MRI in EAS) = 12 > sensitivity = 55%, specificity: 70.5%, PPV: 95.6% - ^68^Ga-DOTA-TATE PET (*n* = 6): + in 4/6 (67%), − in 2/6 (33%, both + on CT) - ^18^F-FDG–PET: 4/4 positive (mapping of disease burden)	Some lesions are better diagnosed by anatomical rather than functional scans, but functional imaging may help in cases where anatomical imaging fails.
[63]	Goroshi et al. (KEM Hospital Mumai, India)	2016	^68^Ga-DOTA-NOC (3–5 mCi)	PET–CT + CECT	12	35.5 (22–45)	42%	Review the performance of ^68^Ga-DOTA-NOC PET–CT and CECT in 12 consecutive EAS patients.		Population: 11 overt cases, 1 remained occult. 13 lesions in 11 patients (true +) ECS localization: - CECT (*n* = 13): + in 12/13 (92%, of which 5 false +), PPV = 71% -^68^Ga-DOTA-NOC PET–CT: + in 9/13 (69%, of which 0 false+ > PPV = 100%)	CECT remains the first-line investigation in EAS localization, and ^68^Ga-DOTA-NOC PET–CT can be added to enhance the PPV of suggestive lesions.
[59]	Venkitaraman et al. (All India Institute of Medical Sciences, India)	2014	^18^F-FDG + ^68^Ga-DOTA-TOC	PET–CT	3	42, 45, 28	unkn	Share experience in successful localization of ectopic EAS in 3 patients with ^68^Ga-DOTA-TOC PET–CT, who later underwent surgical resection and had complete resolution of symptoms.	Typical carcinoid lung (3)	ECS localization - MRI—pituitary (*n* = 3): − in 3/3 - CT—chest *(n = 3*): + in 3/3 - ^18^F-FDG PET–CT *(n = 3*): − in 3/3 - ^68^Ga-DOTA-TOC PET–CT *(n = 3*): + in 3/3 Outcome: 3/3 the complete resolution of symptoms	^68^Ga-DOTA-TOC PET–CT successfully localized ectopic source in all three and also detected the spread to the mediastinal lymph nodes; thus, not only helped in localizing tumors but also influenced surgical decision making.
[70]	Karageorgiadis et al. (National Institutes of Health (NIH), USA)	2015	^18^F-FDG + ^68^Ga-DOTA-TATE	PET–CT + SPECT	7	Median: 13.6 (1–21)	57%	Discussion of localization, work-up, and management of ACTH/CRH co-secreting tumors in children and adolescents.	Metastatic hepatic NET, metastatic pancreatic NET (primary tumor: 2 lobular mass and 1 distal pancreatic tail), thymic carcinoid (3), bronchogenic carcinoid, pancreatoblastoma	ECS localization: - IPSS (*n* = 5): ECS in 4/5 - MRI—pituitary (*n* = 6): - in 4/6, + in 2/6 (hypoenhancing segments suggestive of macroadenomas > 1 TSS) - Abdominal or chest MRI and CT (*n* = 7): + in 7/7 - ^18^F-FDG PET–CT (*n* = 5): + in 4/5 (true positives) - 68Ga-DOTA-TATE PET–CT (*n =* 1): 1/1 uptake	Extremely rare, diagnosis is frequently missed and sometimes confused with CD due to the effect of CRH on the pituitary.
[25]	Koulouri et al. (Wellcome Trust-MRC Institute of Metabolic Science)	2015	^11^C-Met (300–400 MBq)	PET–CT + MRI (SPGR)	2	42 + unknown	unknown	Report experience of functional imaging with ^11^C-Met PET–CT/MRI in the investigation of ACTH-dependent CS.	Small bowel primary NET + primary breast tumor	ECS localization: ^11^C-Met PET–CT: 2/2 Very little uptake in pituitary fossa, but distant sites of metastasis were detected, with subsequent histology confirming ACTH-stained NET	^11^C-Met PET–CT can aid the detection of ACTH-secreting tumors in CS and facilitate targeted therapy: may help inform decision making in EAS with an incidental NFA + source of ECS (including metastases) may be identified.
[71]	Sathyakumar et al. (Christian Medical College, India)	2017	^68^Ga-DOTA-TATE + ^68^Ga-DOTA-NOC + ^18^F-FDG	PET–CT	7 scans (total N = 21)	34 (19–55) (total)	67% (total)	Describe the experience with ECS.	Thymic carcinoid (1), bronchial carcinoid (4)	ECS localization: - CT—chest: (*n* = 17): + in 12/17 (71%), − in 5/17 (29%) - ^68^Ga-DOTA-NOC PET–CT: (*n* = 1): + in 1/1 - ^68^Ga-DOTA-TATE PET–CT: + in 4/5 (80%, of which 3/4 also + on CT), − in 1/5 (20%) - ^18^F-FDG PET–CT: + in 2/2 (100%)	ECS is most commonly seen in association with intrathoracic tumors such as bronchial or thymic carcinoid.
[46]	Deldycke et al. (AZ Sint-Jan Hospital Bruges)	2018	^68^Ga-DOTA-TOC + ^18^F-FDG	PET–CT	1	68	100%	4 case descriptions, highlighting diagnostic challenges and treatment options in paraneoplastic CS.	occult	ECS localization: - BIPSS: ECS in 1/1 - CT: − in 1/1 (no overt tumor; small lung nodule but no malignant aspect, stable upon re-evaluation) - ^18^F-FDG PET–CT: − in 1/1 - ^68^Ga-DOTA-TOC PET–CT: − in 1/1 Outcome: not controlled by SSA/ketoconazole so bilateral adrenalectomy, at follow-up no primary tumor identified	
[65]	Varlamov, Hinojosa and Fleseriu (Oregon Health & Science University, USA)	2019	^68^Ga-DOTA-TATE + ^111^In + ^18^F-FDG	PET–CT, SPECT, pituitary MRI, body CT or MRI	6	54.6 (±16.1)	17%	Report on 6 consecutive patients with confirmed active and occult ECS who underwent ^68^Ga-DOTA-TATE PET–CT.	Occult (4), pancreatic NET (1)	ECS localization: - IPSS (*n* = 4): ECS in 4/4 - pituitary MR (*n =* 6): − in 3/6, + in 3/6 (2x 4 mm + 1x 3 mm > 1 patient TSS but later D/pancreatic tumor) - CT body (*n =* 6): − in 1/6, + in 5/6 - ^18^F-FDG PET–CT (*n =* 5): − in 2/5, + in 3/5(of which 2 false +) - ^68^Ga-DOTA-TATE PET–CT (*n =* 6): − in 4/6, + in 2/6 Outcome: 2 occult: medical therapy with ketoconazole/mifepristone, 2 bilateral adrenalectomy	Center experience demonstrates a lower than previously reported ^68^Ga-DOTA-TATE PET–CT sensitivity for ECS, especially in occult lesions. We suggest that data on this tracer in ECS is subject to publication bias and false – are likely underreported; its diagnostic value needs further study. ^68^Ga-DOTA-TATE PET–CT suggestive of ECS source in one overt case (seen on CT) and did not help identify a culprit lesion in five occult lesions.
[60]	Wannachalee et al. (3 tertiary referral centers: University of Michigan, Mayo Clinic Rochester + The University of Texas MD Anderson Cancer Center, USA)	2019	^68^Ga-DOTA-TATE	PET–CT	28	50 (38–64)	21%	Determine the efficacy for ECS localization and clinical benefit of ^68^Ga-DOTA-TATE PET–CT.	Bronchial (5), thymic (1), pancreatic (1), metastatic NET of unknown origin (1)	Population: 28 ECS: 17/28 identified, 15/28 occult ECS localization: - ^68^Ga-DOTA-TATE PET–CT: + in 11/17 (65%; 7/11 solitary and 4/11 metastatic), − in 6/17 1 false +: adrenocortical hyperplasia 1 false-: lever metastasis First modality to localize in 2/17 Outcome: Follow-up (*n* = 11) ^68^Ga-DOTA-TATE PET–CT: 7/11 (64%) changes in clinical management (4/7 no change) > identified 9 new metastatic foci and 3 recurrent tumors (5 solitary bone, 3 pancreatic, 1 abdominal lymph)	^68^Ga-DOTA-TATE PET–CT is sensitive in detecting primary and metastatic ECS, often identifies occult tumors after conventional imaging, and impacts clinical care in the majority of patients. A somewhat lower accuracy in this series might reflect selection bias for difficult cases at referral centers, with a high prevalence of occult ECS.
[64]	Ceccato et al. (University Hospital of Padova, Italy)	2020	^68^Ga-DOTA-TOC + ^68^Ga DOTA-NOC (2 MBq/kg)	PET–CT	18		44%	Study the diagnostic accuracy of CT with ^68^Ga-SSTR PET–CT in localizing ACTH-secreting tumors in patients with EAS.	Bronchial carcinoma (11), SCLC (2), pancreatic NET (1)	Population: 18 ECS cases (16 primary + 2 recurrent neoplasms) > 8 overt and 10 occult: 6/10 covert after careful follow-up and 4/10 remained occult ECS localization: - BIPSS (*n =* 10): ECS in 10/10 - CT (*n* = 18) + in 11/18 (61%), − in 7/18 (39%) - ^68^Ga-SSTR PET–CT: + in 12/28, − in 16/28 *Overt cases:* (*n* = 8) - de novo overt ECS: CT + in 4/6, ^68^Ga-SSTR + in 6/6 - recurrent/relapse ECS: CT + 2/2, ^68^Ga-SSTR + in 1/2 Occult cases (*n* = 10, 6/10 localized after careful FU) - 1 + on CT, never showed pathological uptake ^68^Ga-SSTR - 1 + on ^68^Ga-SSTR, not found on conventional radiology *Baseline:* - CT (*n* = 16): + in 4/16, 1/16 inconclusive, − in 12/16 (of which 2+ on ^68^Ga-SSTR) - MRI (*n* = 6): - in 6/6 - ^68^Ga-SSTR PET–CT (*n* = 16): + in 6/16, − in 10/16 *Follow-up:* - CT (*n* = 15): + in 7/15, − in 8/15 (of which 1 + on 68Ga) - MRI (*n* = 3): + in 2/3, − in 1/3 - ^68^Ga-SSTR PET/CT (*n* = 12): + in 6/12, − in 6/12 (of which 2+ on CT)	^68^Ga-SSTR PET–CT is useful in localizing EAS, especially to enhance positive prediction of the suggestive CT lesions and to detect occult neoplasms. However, it presents a considerable number of indeterminate/false+ images that need careful interpretation. Nuclear and conventional imaging should be repeated during follow-up (occult + overt).
[61]	Bélissant et al. (Hôpital Tenon APHP and Sorbonne University)	2020	^68^Ga-DOTA-TOC (1.2–2 MBq/kg)+ ^18^F-FDG + F-DOPA	PET–CT + CECT	19	Range: 26–71	26%	Report experience with ^68^Ga-DOTA-TOC PET–CT in localizing causal NET in case of initial but also recurrent paraneoplastic Cushing’s syndrome, and its clinical impact.		ECS localization: *Primary NET (sensitivity/accuracy):* - CECT: 2/7 (29%)/3/9 (33%) - ^18^F-FDG PET–CT: 1/6 (17%)/1/7 (14%) - DOPA PET–CT: 0/6 (0%)/0/6 (0%) - ^68^Ga-DOTA-TOC PET–CT: 4/8 (50%)/5/9 (55%) *Persistence: (n = 3) or recurrence (n = 4) of PCS* (sensitivity per patient/sensitivity per lesion/accuracy per lesion): - CECT: 2/7 (29%)/2/9 (22%)/2/9 (22%) - ^18^F-FDG PET–CT: 0/3 (0)/0/3 (0)/0/4 (0) - DOPA PET–CT: 0/1 (0)/0/1 (0)/0/1 (0) - ^68^Ga-DOTA-TOC PET–CT:6/7(86%)/9/10 (90%)/9/11(82%) Outcome: ^68^Ga-DOTA-TOC PET–CT clinical impact: 3/13 (23%) primary NET and DOTA-TOC PET/CT alone had a clinical impact in 4/7 (57%) persistent patients.	^68^Ga-DOTA-TOC PET–CT seems to be a valuable tool for the detection of NET responsible for persistent/recurrent paraneoplastic Cushing’s syndrome after surgery (more effective than the detection of causal tumor initial PCS). Also valuable for staging when primary NET is easily found > help localize additional lesions (small lymph nodes).
[29]	Walia et al. (Post Graduate Institute of Medical Education and Research, India) **Prospective**	2021	^68^Ga-tagged CRH (111–185 MBq)	PET–CT	3	unknown	33%	Evaluate the role of ^68^Ga-CRH PET–CT for the evaluation and management of ACTH-dependent CS.	Bronchial NET (2), pancreatic NET (1)	ECS localization: - BIPSS (*n* = 1): central gradient - MRI—pituitary (*n* = 3): − in 2/3, + in 1/2 (suspicious lesion 3 × 4 mm) - 68Ga-CRH PET–CT: detection of primary NET = 3/3 uptake tracer in sella: + in 1/3 (diffuse), − in 2/3 Outcome: 1 resolution after surgery and 1 died before surgery	^68^Ga-CRH PET–CT represents a novel, noninvasive molecular imaging, targeting CRH receptors that not only delineate corticotropinoma and provide the surgeon with valuable information on intraoperative tumor navigation but also help in differentiating a pituitary from an extra-pituitary source of ACTH-dependent CS.
[51]	Zisser et al. (Vienna general hospital, Italy)	2021	^18^F-FDG (5 MBq/kg) + ^18^F-DOPA (3 MBq/kg) + ^68^Ga-DOTA-NOC (3 MBq/kg)	PET–CT	18	51 ± 18 (confirmed: 43±17; occult: 57±16)	58% (con: 62%, occ: 50%)	Evaluate the diagnostic feasibility of ^18^F-FDG, ^18^F-DOPA, ^68^Ga-DOTA-NOC PET–CT in ECS.	Pulmonary NET, SCLC, papillary thyroid carcinoma	Population: 18 ECS: confirmed (*n* = 7; all visible on conventional imaging), highly suspected without histology (*n* = 1), and occult (*n* = 10). ECS detection: - CT—chest (*n* = 12): + in 6/12 (50%; 5/6 true+ and 1/6 false+) - MRI—chest (*n* = 1): + in 0/1 - CT—abdomen (*n* = 10): + in 0/10 - MRI—abdomen (*n* = 5): + in 0/5 - ^18^F-FDG PET–CT (*n* = 11): + in 5/11 (3/5 true+, 2/5 false+) - ^18^F-DOPA PET–CT (*n* = 11): + in 3/11 (3/3 true+, 0 false+) - ^68^Ga-DOTA-NOC PET–CT (*n* = 8): + in 4/8 (3/4 true+, 1 false+) *Confirmed cases: (n = 7)* - ^18^F-FDG PET–CT: true+ in 3/6 (50%), false- in 3/6 (50%) sensitivity = 50%, specificity = 60% (3/5), PPV = 60%, NPV = 50%, accuracy = 55% (6/11) - ^18^F-DOPA PET–CT: true+ in 3/4 (75%), false− in 1/4 (25%) sensitivity = 75%, specificity = 7/7 (100%), PPV 100%, NPV 88%, accuracy = 90% (10/11), - ^68^Ga-DOTA-NOC PET–CT: true+ in 3/3, false− in 0 sensitivity = 100%, specificity = 80% (4/5), PPV = 75%, NPV = 100%, accuracy = 88% (7/8)	^68^Ga-DOTA-NOC and ^18^F-DOPA PET–CT superior results compared to ^18^F-FDG. The sensitivity might be influenced by the etiology of the underlying tumor. ^68^Ga-DOTA-NOC is a highly recommendable tracer for tumor detection in ECS, and F-DOPA has promising results (especially specificity) but needs further assessment; FDG can be complementary.
[31]	Novruzov et al. (University College London Hospital, UK)	2021	^68^Ga-DOTA-TATE (120–200 MBq)	PET–CT	8	50 ± 23 (14–78)	25%	Investigate the utility of ^68^Ga-DOTA-TATE PET–CT in patients with suspected pituitary pathology.	Bronchial carcinoma (3 of which 2 typical), small bowel NET (1), pancreatic NET (1)	Population: ECS: 6 de novo and 2 suspected recurrent CD ECS detection: - ^68^Ga-DOTA-TATE PET–CT: + in 6/8 (75%), −in 2/8 (25%) + = 5/6 (83%) de novo and 1/2 (50%) recurrent First to localize in 1/5 (terminal ileum) 2 cases occult: not shown on any imaging modality > 1/2 detected on EUS (pancreatic NET) and 1/2 occult	^68^Ga-DOTA-TATE is useful in CS for the detection of ectopic sources of ACTH production, especially when anatomic imaging is negative.
[56]	Hou et al. (Peking Union Medical College Hospital, China)	2021	^18^F-FDG (5.55 MBq/kg) + ^68^Ga-DOTA-NOC (111–148 MBq) + ^99m^Tc	PET–CT/SPECT-CT	24	38 ± 17 (9–72)	54%	Evaluate the usefulness of ^18^F-FDG PET–CT for differentiating ectopic ACTH-secreting lung tumors from tumor-like pulmonary infections in patients with ECS.	Lung tumors (18, of which 12 are typical, 5 atypical, 1 SLCL)	Population: 24 cases with pulmonary CT nodules and occult source of ECS (18 lung tumors and 6 pulmonary infections) ECS detection: - MRI—pituitary: − in 18/24 (75%), - ^18^F-FDG PET–CT: SUVmax of infections higher than tumors, SUVmax > 4.95 = differentiating 75% sensitivity and 94% specificity. - SRI: (*n* = 15 of which 6 ^99m^Tc + 9 ^68^Ga): lung tumors: sensitivity = 6/11 (55%) and spec = 50% lung infections: + in 2/4 (50%; 3/4 ^99m^Tc)	Pulmonary infections exhibit higher ^18^F-FDG uptake than ACTH-secreting lung tumors. An SUVmax cut-off value of 4.95 may be useful for differentiating the two conditions. SRI may not be an effective tool for differentiating the two conditions (low specificity).
[32]	Ding et al. (Peking Union Medical College Hospital, China)	2022	^68^Ga-pentifaxor	PET–CT	1	39	100%	Investigate the performance of ^68^Ga-pentixafor in CS.		Case of metastatic ECS: multiple hypermetabolic lesions, no pituitary uptake, increased adrenal uptake	Promising in the DD of both ACTH-independent and ACTH-dependent CS. Activated CXCR4 molecular signaling along the pituitary–adrenal axis was found in patients with Cushing’s disease.
[57]	Zhang et al. (The First Affiliated Hospital of Sun Yat-Sen University, China)	2022	^18^F-FDG (5.55 MBq/kg) + ^68^Ga-DOTA-NOC (111–185 MBq)	PET–CT	68	51 [42,43,44,45,46,47,48,49,50,51,52,53,54,55,56,57,58,59,60,61] (19–72)	44%	Compare the diagnostic efficacies of ^18^F-FDG and ^68^Ga-DOTA-NOC PET–CT in EAS.	Anterior mediastinal/thymic (21), bronchial carcinoid (9), pancreatic NET (10), paraganglioma/pheochromocytoma (3), medullary thyroid carcinoma (2), olfactory neuroblastoma (2), occult (21)	Population: 68 cases: 37/68 (54%) for tumor localization, 31/68 (46%) to evaluate tumor load/metastasis (staging) ESC detection: Localization group (*n* = 37): primary tumor detected in 18/37 (49%) Scan-based: (*n* = 37) - ^18^F-FDG PET–CT: true+ in 7/37 (19%), false+ in 8/37 (22%) and false- in 1/37 (3%) - ^68^Ga-DOTA-NOC PET–CT: true+ in 17/37 (46%), false+ in 1/37 (3%) and false- in 1/37 (3%) *Lesion-based: (n = 29):* - ^18^F-FDG PET–CT: true+ in 7/29 (24%), false+ in 9/29 (31%) and false− in 1/37 (3%) - ^68^Ga-DOTA-NOC PET–CT: true+ in 17/29 (59%), false+ in 1/29 (3%) *In overt EAS (n = 18)* - ^18^F-FDG PET–CT: sensitivity = 7/18 (39%) - ^68^Ga-DOTA-NOC PET–CT: sensitivity = 17/18 (94%) 6/18 (33%) + on both, 11/18 (61%) + only in 68Ga, 1/18 (6%) only + in FDG Staging group (*n* = 31): 11/31 after primary tumor resection with 286/292 confirmed as tumor lesions. 28/31 (90%) patients with detected tumor lesions. *Scan-based:* - ^18^F-FDG PET–CT: true+ in 26/31 (84%), false+ in 4/31 (13%) - ^68^Ga-DOTA-NOC PET–CT: true+ in 21/31 (68%), false+ in 3/31 (10%) *Lesion-based:* -^18^F-FDG PET–CT: true+ in 274/292(94%), false+ in 4/292(1%) - ^68^Ga-DOTA-NOC PET–CT: true+ in 160/292 (55%), false+ in 3/392 (1%) 148/286 (52%) + on both exams; 126/286 (48%) + only FDG, 12/286 (4%) + only 68Ga	^68^Ga-DOTA-NOC PET–CT imaging may be more suitable than ^18^F-FDG for identifying primary tumors in ECS, while ^18^F-FDG PET–CT may be more advantageous than ^68^Ga-DOTA-NOC for patients with suspected metastasis.

^11^C-5-HTP = ^11^C-5-hydroxytryptophan, ^99m^Tc = Technetium-99m; CECT = contrast-enhanced CT; L-OCT = low-dose ocreotidescan, H-OCT = high-dose ocreotidescan. * Given as % male.

## Appendix B

**Table A2:** Diagnostic accuracy of molecular imaging in ectopic Cushing’s syndrome.

		^18^F-FDG (16 Articles)	^68^Ga-SSTR (16 Articles)	^68^Ga-DOTA-TOC (5 Articles)	^68^Ga-DOTA-TATE (7 Articles)	^68^Ga-DOTA-NOC (4 Articles)	^68^Ga-Pentixafor (1 Article)	^68^Ga-CRH (1 Article)	^18^F-DOPA (3 Articles)	^11^C-Met (1 Article)	^11^C-5-HTP (1 Article)
All	True +	46% (75/164)	59% (109/185)	65% (17/26)	49% (29/59)	62% (51/82)	100% (1/1)	100% (3/3)	32% (7/22)	100% (2/2)	100% (1/1)
	False +	22% (23/106)	6% (7/124)	13% (1/8)	3% (1/34)	6% (5/82)		33% (1/3)	0% (0/9) *		
Overt	True +	54% (47/87)	74% (73/98)	70% (7/10)	70% (29/41)	77% (30/39)		100% (3/3)	35% (7/20)	100% (2/2)	100% (1/1)
	False +	60% (3/5)	3% (2/78)	20% (1/5)	3% (1/34)	0% (0/39)		33% (1/3)	0% (0/9)		
PET	True +	47% (26/55)	67% (4/6)		67% (4/6)				47% (7/15)		100% (1/1)
	False +	24% (4/17)							0% (0/2)		
PET–CT	True +	45% (49/109)	59% (105/179)	65% (17/26)	47% (25/53)	62% (51/82)	100% (1/1)	100% (3/3)	0% (0/7)	100% (2/2)	
	False +	20% (20/101)	6% (7/124)	13% (1/8)	3% (1/34)	6% (5/82)		33% (1/3)	0% (0/7)		
De novo	True +	38% (49/130)	49% (74/151)	58% (11/19)	49% (28/57)	49% (29/59)	100% (1/1)	100% (3/3)	33% (7/21)	100% (2/2)	100% (1/1)
	False +	25% (19/75)	4% (4/101)	13% (1/8)	3% (1/34)	3% (2/59)		33% (1/3)	0% (0/2) *		
Recurrent	True +	76% (26/34)	65% (34/52)	86% (6/7)	50% (1/2)	68% (21/31)			0% (0/1)		
	False +	12% (4/34)	10% (3/31)			10% (3/31)			0% (0/1)		

Given as % (total patients positive/total tested patients). * Zemskova et al. found 11% false positive lesions but did not provide the number of patients [68].
